# Application of Magnetic Nanoparticles Coated with Crosslinked Zwitterionic Poly(ionic liquid)s for the Extraction of Oligonucleotides

**DOI:** 10.3390/ma14123146

**Published:** 2021-06-08

**Authors:** Łukasz Nuckowski, Krzysztof Dzieszkowski, Zbigniew Rafiński, Sylwia Studzińska

**Affiliations:** 1 Chair of Environmental Chemistry and Bioanalytics, Faculty of Chemistry, Nicolaus Copernicus University in Toruń, 7 Gagarin Str., PL-87-100 Toruń, Poland; kowalska@chem.umk.pl; 2 Chair of Organic Chemistry, Faculty of Chemistry, Nicolaus Copernicus University in Toruń, 7 Gagarin Str., PL-87-100 Toruń, Poland; dzieszko@doktorant.umk.pl (K.D.); payudo@chem.umk.pl (Z.R.)

**Keywords:** antisense oligonucleotides, magnetic nanoparticles, poly(ionic liquid)s, magnetic dispersive solid-phase extraction, serum samples

## Abstract

Magnetic nanoparticles coated with zwitterionic poly(ionic liquid)s were applied for dispersive solid-phase extraction of oligonucleotides. The materials were synthesized by miniemulsion copolymerization of ionic liquids and divinylbenzene on magnetic nanoparticles. The functional monomers contain a positively charged imidazolium ring and one of the anionic groups: derivatives of acetate, malonate, or butyl sulfonate ions. Adsorption of unmodified DNA oligonucleotide on obtained materials was possible in ion-exchange (IE) and hydrophilic interactions (HI) mode. The adsorption in IE was possible at low pH and was almost complete. The adsorption in HI mode required the usage of appropriate addition of organic solvent but did not provide full adsorption. Studies on the desorption of the analytes included determining the impact of ammonium acetate concentration and pH and organic solvents addition on the recovery. The material containing acetic fragments as an anionic group was selected for the final procedure with the use of 10 mM ammonium acetate (pH = 9.5)/methanol (50/50, *v*/*v*) as an elution solution. The magnetic dispersive solid-phase extraction procedure was tested for the oligonucleotides with various modifications and lengths. Moreover, it was applied to extract DNA oligonucleotide and its synthetic metabolites from enriched human plasma without any pre-purification, with recoveries greater than 80%.

## 1. Introduction

Oligonucleotides (OGNs) are short analogs of nucleic acids, so they are built from nucleobases and sugar-phosphate backbone. One strand of human DNA is made up of several billion nucleotides when OGN length does not exceed a few dozens of it. An example of an important naturally occurring OGNs are microRNA, which plays a significant role in organism functions [1]. The participation of these molecules in biological processes is important for oncogenesis, which is why they are studied as a diagnostic and therapeutic factor [1,2,3]. Synthetic OGN often has chemical modifications and give promising results as therapeutics in many types of diseases, including cancer, metabolic, inflammatory, infectious, and neurological ones [4,5]. Whatever the reason for the interest in a specific type of OGN their analysis has a great role; thus, sensitive methods are needed. Nowadays, high-performance liquid chromatography (HPLC) is the most widely used technique in this field [6,7,8]. Analysis of OGN is preceded by the sample preparation step [9]. Among the techniques of sample cleaning, solid-phase extraction (SPE) with various adsorbents is dominant [9].

Commercially available anion-exchange resigns, such as Clarity OTX^®^ and Oasis WAX^®^, are one of the most popular materials used during OGN SPE. Optimized procedures that use these adsorbents give high recovery values, usually in the range of 60–90% [10,11,12,13,14,15]. However, the protocols often required additional preliminary cleaning step with the application of lysis buffer to disrupt protein binding [12,15]. Moreover, some SPE procedures reported in the literature assume the application of a high concentration of salts, so they are not compatible with mass spectrometry (MS) detection or required additional desalination step [9,16].

It is worth highlighting that a classical SPE is nowadays replaced by its miniaturized forms. They, among others, minimize toxic and hazardous organic solvent consumption, reduce the sample volume, and maximize extraction efficiency at the same time [17,18]. One of the miniaturized dispersive SPEs involves the use of magnetic particles modified or coated with different adsorbents [19].

Functionalized magnetic particles, modified in different manner are intensively used in nucleic acids separation [20,21]. This approach allows for extraction of DNA or RNA, avoiding centrifugation steps. Automation of whole process is easy and nucleic acids can be extracted from higher volumes of samples [20]. Different types of magnetic particles are used, usually coated with silica, cellulose, or synthetic or biopolymers [20]. Nucleic acids can randomly interact with the modified bead surface, by hydrogen, ionic, and many other interactions. Adsorbed DNA is usually used for further proceedings. If desired, it may be eluted by using a higher ionic strength, pH changes, heating, etc. [20].

Strong anion-exchange magnetic particles (SAX MP) were used for the extraction of short interfering RNA (siRNA) molecules by Ye and Beverly [16]. The authors tested two commercial SAX MP surface-modified with quaternary functional groups. The elution of analytes was possible with a high (1 M) concentration of salts. The application of volatile salts (triethylammonium bicarbonate, ammonium bicarbonate, and ammonium chloride) helped to solve the problem of incompatibility with MS detection. The recovery was dependent on OGN and SAX MP type. Moreover, there were differences in the extraction recovery of analytes with different lengths. These observations suggest that the extraction protocol must be developed every time and assessed empirically [16].

In our previous studies, we developed the method of OGNs extraction with the usage of poly(ionic liquid)s [22]. The dispersive micro-solid-phase extraction was characterized by high recoveries and good repeatability. A drawback of this procedure was the application of centrifuge as a phase separation tool [22]. To resolve this limitation, the current research aimed at the application of magnetic nanoparticles (MNP) coated with crosslinked zwitterionic poly(ionic liquid)s. Due to the character of these coatings adsorption in ion-exchange and hydrophilic interaction modes were tested. The desorption studies were carried out by changing various conditions, namely the salt concentration in the elution solution, its pH, and the percentage of the organic reagent, to obtain the highest recovery value. Developed magnetic dispersive solid-phase extraction (MDSPE) procedure was tested for OGNs with various chemical modifications and lengths. Furthermore, it was used to extract OGN and its synthetic metabolites from enriched serum samples before their analysis by ultrahigh-performance liquid chromatography with diode array detection (UHPLC–DAD).

## 2. Materials and Methods

### 2.1. Materials and Reagents

Lyophilized standards of OGN were bought from Eurogentec (Seraing, Belgium) and Sigma-Aldrich (St. Louis, MI, USA). Sequence, type of modification and molecular mass of each used OGN was presented in Table 1. What is important, each phosphate group and sugar in the sequence was modified. To the lyophilized OGNs appropriate volume of water was added to obtain 0.1 mM standard solutions. Further dilution with water was performed to obtain working solutions in appropriate concentrations.

Methanol (MeOH) with HPLC gradient grade purity, acetone (ACE), and divinylbenzene (DVB) were purchased from Merck (Darmstadt, Germany). Sodium hydroxide (NaOH), hydrochloric acid (HCl), 25% ammonia (NH_3_) solution, iron(III) chloride hexahydrate (FeCl_3_ × 6H_2_O), iron(II) sulfate heptahydrate (FeSO_4_ × 7H_2_O), toluene, ethanol, disodium hydrogen phosphate dodecahydrate (Na_2_HPO_4_ × 12H_2_O) sodium dihydrogen phosphate (NaH_2_PO_4_) and potassium persulfate (K_2_S_2_O_8_) were purchased from POCH S.A. (Gliwice, Poland). Acetic acid (≥99.7%) (AcOH), ammonium acetate (NH_4_OAc), *N*-vinylimidazolium, ethyl bromoacetate, diethyl bromomalonate, 1,4-butane sultone, and sodium dodecyl sulfate (SDS) were purchased from Sigma-Aldrich (St. Louis, MO, USA). HPLC-grade acetonitrile (ACN), chloroform, pentane, and diethyl ether were purchased from J.T. Baker (Center Valley, PA, USA). The 3-(methacryloxy)propyltrimethoxysilane (MPS) was purchased from Fluorochem Ltd. (Hadfield, UK). Deionized water was obtained from a Milli-Q system (Millipore, El Paso, TX, USA).

### 2.2. Apparatus and Conditions

#### 2.2.1. Characterization of Polymerizable ILs and MNPs

The Bruker Avance III 700 MHz spectrometer (Bruker, Billerica, MA, USA) was used for recording a nuclear magnetic resonance (NMR) spectra. The Alpha FTIR Spectrometer (Bruker) with an attenuated total reflection (ATR) mode was used for recording infrared (IR) spectra in the 4000–400 cm^−1^ region. All spectra were provided in Supplementary Materials (Figures S1–S13). Using a Vario Macro CHN Element Analyzer (Elementar Analysen Systeme GmbH, Hanau, Germany) elemental analysis (C, H, N) was carried out. Transmission electron microscopy (TEM) observations were performed by using a Tecnai F20 X-Twin instrument (FEI Europe B.V., Eindhoven, The Netherlands).

#### 2.2.2. Chromatographic Method

Mobile phase A (10 mM NH_4_OAc) and mobile phase B (MeOH) were used during the chromatographic separations in the UltiMate^®^ 3000 Binary Rapid Separation liquid chromatography system equipped with a DAD-3000RS Diode Array Detector (Dionex, Sunnyvale, CA, USA). The detection wavelength was λ = 260 nm. During the study, ACE Excel C18 (1.7 µm, 100 × 2.1 mm) column (Advanced Chromatography Technologies Ltd., Aberdeen, UK) was used. The temperature of the autosampler and the column was 30 °C. The Chromeleon 7 chromatography data system was used for data collecting. The injection volume was 2 µL. The flow rate was equal to 0.15 mL min^−1^. The chromatographic separations were carried out in gradient elution mode. To analyze the samples with one compound, the gradient elution program was linear: 5–60% of solvent B in 5 min. For the separation of DNA-20, DNA-18, and the DNA-16 mixture, a gradient elution program from 5 to 20% of solvent B in 5 min and from 20 to 25% of solvent B in 10 min was run.

### 2.3. Synthesis of Polymerizable IL and MNPs

The polymerizable ionic liquids (IL) were synthesized according to the procedures described by Zhao et al. [23]. The Fe_3_O_4_ and Fe_3_O_4_-MPS nanoparticles were prepared according to the procedures described by Chen et al. [24]. The Fe_3_O_4_-MPS was coated with crosslinked poly(ionic liquid)s with the procedure described by Yang et al. [25] that was modified for our purposes. All used procedures are described in detail in the Supplementary Materials.

### 2.4. Adsorption and Desorption of OGN

The adsorption of OGN was performed in two modes: ion-exchange (IE) mode and hydrophilic interactions (HI) mode.

In the IE, the amount of 2.0 mg of coated nanoparticles (MNP-Ac, MNP-Sul, and MNP-Mal) was conditioned by mixing with 100 µL MeOH in an Eppendorf tube. The sorbent was separated from the solution, using a strong magnet and the supernatant was removed. Next, the procedure was repeated with 100 µL of water or 10 mM solution of NH_4_OAc at an appropriate pH (3.5, 4.5, 5.5, or 6.5). Then, 50 µL of 5 µM OGN was mixed with 50 µL of water or NH_4_OAc at the same pH, and this mixture was added to the conditioned adsorbent and vortexed, and the phases were magnetically separated. The supernatant was removed, centrifuged (10 min, 14,462× *g*), and analyzed.

In the HI mode, the amount of 2.0 mg of coated nanoparticles was conditioned by mixing with 100 µL of appropriate water/organic solvent (ACN or ACE) mixture in an Eppendorf tube. The solid phase was separated using a magnet, and the supernatant was removed. Next, 50 µL of 5 µM OGN was mixed with an appropriate amount of organic solvent (ACN or ACE), and this mixture was added to the conditioned adsorbent and vortexed; then, the phases were magnetically separated. The supernatant was removed, partially evaporated by using a CentriVap vacuum concentrator (Labconco, Kansas City, MO, USA) at 40 °C to 10 μL, filled to 50 µL, centrifuged (10 min, 14,462× *g*), and then analyzed. Detailed conditions are presented in Section 3.3.2.

The developed procedure with three different sample weights were used for determination of sorption capacity. This parameter was calculated by using Equation (1), where *Q* is the sorption capacity (µmol g^−1^), *c*_0_ is the OGN concentration before adsorption (µmol mL^−1^), *c* is the OGN concentration after adsorption, *V* is the volume of OGN solution used during procedure (mL), and *m* is the mass of the weight of the adsorbent (g).

(1) $$Q=\frac{\left(c_{0}-c\right)\times V_{m}}{m}$$

The studies of influence of adsorption time on its effectiveness were performed for DNA-20 OGN, following the procedure for adsorption in IE mode, but for five different adsorption times (1, 5, 10, 30, and 60 min).

The desorption of OGN was performed by vortexing sample weight of the coated nanoparticles with 50 µL of elution solution (see Section 3.3) for 30 min. The nanoparticles were separated by a magnet, the liquid phase was transferred into another Eppendorf tube, centrifuged (10 min, 14,462× *g*), and the supernatant was analyzed. The studies of influence of desorption time on the recovery was performed for DNA-20 OGN, following the developed procedure for desorption in IE mode, but for five different desorbtion times (1, 5, 10, 30, and 60 min).

### 2.5. Fortification and Preparation of Serum Samples

Human serum was enriched with an appropriate volume of working solution of an OGN mixture (DNA-16, DNA-18, and DNA-20). Next, it was diluted with water. In each experiment, final plasma dilution was 1:5. The samples fortified by this method were prepared by using the final procedure (Section 3.4) without any additional pretreatment.

### 2.6. Chromatographic Method Validation

For determination of recovery of OGNs, serum samples were spiked to a concentration of 5 μM of each analyte (DNA-16, DNA-18, and DNA-20). The recovery values were calculated by comparison of peak areas for samples obtained after extraction with final procedure, and standard solutions of the same concentration. The matrix effect was determined by following a typical procedure described in the literature [26], by comparison of the peak area for a standard sample with the peak area of the equivalent concentration of the analyte in a blank matrix sample spiked post-extraction. The matrix effect was included in the calculation of OGNs recoveries from spiked serum samples. Calibration curves were plotted based on standard solutions at seven concentrations (1.25, 2.5, 5.0, 7.5, 10.0, 15.0, and 20.0 µM). The coefficient of determination (R^2^) showed the linearity for the calibration curve. A relative standard deviation of peak area for 10 injections in one day for concentration (for 1.25, 7.5, and 15 µM) was calculated to the determination of intraday precision. A relative standard deviation of peak area for 5 injections at three different concentrations (for 1.25, 7.5, and 15 µM) during the first, third, and seventh day of the experiment was calculated to the determination interday precision (repeatability). The limit of detection (LOD) and the limit of quantification (LOQ) were calculated based on Equations (2) and (3), respectively. In these equations, *s* is the standard deviation of the calibration curve intercept, and *a* is the slope of the calibration curve.

(2) $$LOD=\frac{3\times s}{a}$$

(3) $$LOQ=\frac{10\times s}{a}$$

## 3. Results and Discussion

### 3.1. Synthesis and Characterization of ILs and MNPs

**Figure 1:**
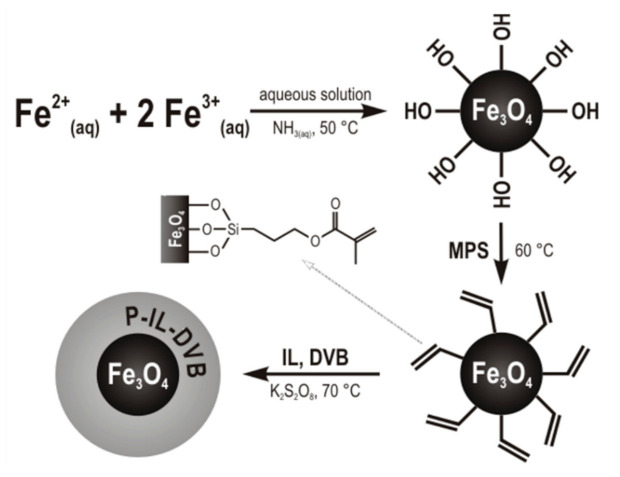
General scheme of synthesis of magnetic nanoparticles coated with crosslinked zwitterionic poly(ionic liquid)s.

**Figure 2:**
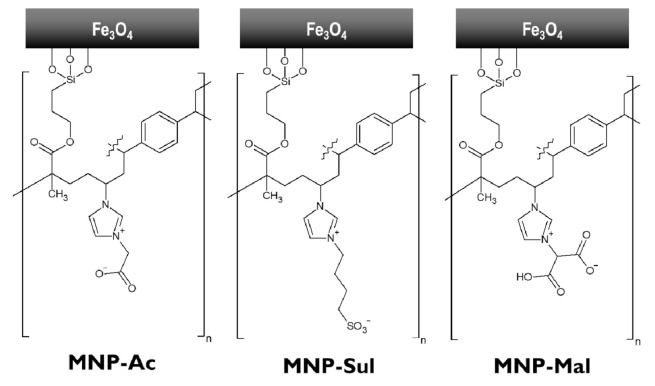
Schematic structures of magnetic nanoparticles coated with crosslinked zwitterionic poly(ionic liquid)s obtained in this study.

The magnetic nanoparticles coated with crosslinked zwitterionic poly(ionic liquid)s were prepared by following the general scheme presented in Figure 1. The schematic structures of the MNPs are presented in Figure 2.

Previous studies performed in our group [22] showed promising results of the application of zwitterionic, imidazolium-based crosslinked poly(ionic liquid)s in the extraction of OGNs. Thus, we decided to test three MNPs covered with different zwitterionic coatings. The first one (MNP-Ac) is analogical to this, which gave the best results in our previous paper [22]. In the MNP-Sul, a carboxyl group was replaced by a sulfonic group, often present in the structures in the ligands of zwitterionic stationary phases. The last material, MNP-Mal, has a coating with the malonic ligand. An additional carboxyl group was intended to increase the electrostatic repulsion in the elution stage.

#### 3.1.1. Synthesis and Characterization of ILs

Three different polymerizable ILs with a vinyl functional group were synthesized. They were used as functional monomers in the copolymerization coating of MNPs. The structures of ILs were confirmed by ^1^H NMR and FT-IR. Supplementary Materials Figures S1, S3, and S5 show ^1^H spectra for three synthesized monomers: EtAcviimBr, Sulviim, and EtMalviimBr. Signals over +7.25 ppm in the Sulviim spectrum (Supplementary Materials Figure S3), and over +7.5 ppm in EtAcviimBr and EtMalviimBr spectra (Supplementary Materials Figures S1 and S5) correspond to the presence of the imidazolium protons. The presence of vinyl protons was confirmed by appropriate signals: +5.50 ppm and in the range of +5.9–7.4 ppm for EtAcviimBr (Supplementary Materials Figure S1); in the range of +5.0–7.2 ppm for Sulviim (Supplementary Materials Figure S3); and in the range of +5.4–6.0 ppm and +7.33 ppm for EtMalviimBr (Supplementary Materials Figure S5). In the EtAcviimBr 1H NMR spectrum, the signal at +5.54 ppm corresponds to the methylene protons between the imidazolium ring and the ester group (Supplementary Materials Figure S1). The signal at +7.25 ppm in the EtMalviimBr corresponds to the tertiary hydrogen atom (Supplementary Materials Figure S3). In both spectra, EtAcviimBr and EtMalviimBr, signals with chemical shift values below +5.0 ppm confirm the presence of alkyl protons (Supplementary Materials Figures S1 and S3). The signals below +4.5 ppm in the Sulviim spectrum correspond to the presence of a butylene protons between the imidazolium and sulfonate groups (Supplementary Materials Figure S5).

#### 3.1.2. Synthesis and Characterization of MNP

The Fe_3_O_4_ particles were first prepared (Figure 1). Their TEM image was shown in Supplementary Materials Figure S14A. The diameter of these nanoparticles is in the range from 9 to 20 nm, with an average size of 13 nm and a relative standard deviation of 20% (*n* = 50) (Supplementary Materials Figure S15). Next, the Fe_3_O_4_ surface was modified with MPS (Figure 1). Supplementary Materials Figure S7 shows FTIR spectra of the MPS-functionalized magnetic nanoparticles. The characteristic absorption bands at and 1629 cm^−1^ are present due to C=C stretching tensions of the MPS ligand. The bands at 1688 and 1169 cm^−1^ are present due to the C=O and C–O stretching vibrations, respectively (Supplementary Materials Figure S7).

The Fe_3_O_4_-MPS were coated with poly(ionic liquid)s crosslinked with divinylbenzene. The coating was performed by miniemulsion polymerization of the synthesized ILs in the presence of DVB as a cross-linker and K_2_S_2_O_8_ as an initiator (Figure 1). Changes in the FT-IR spectra confirm the progress of the polymerization reaction. Peaks characteristic of stretching tensions of C=C in the range of 1650–1670 cm^−1^ in the monomer spectra (Supplementary Materials Figures S2, S4, and S6), disappeared in the coated MNP spectra (Supplementary Materials Figures S9, S11, and S12). The incorporation of IL in polymer structure was proved by the presence of nitrogen confirmed in elemental analysis. The nitrogen content for MNP-Ac, MNP-Sul, and MNP-Mal was 0.39, 0.86, and 0.65%, respectively.

Coating of MNP-Ac and MNP-Mal with zwitterionic, crosslinked poly(ionic liquid)s was performed in two steps. In the first stage, the Fe_3_O_4_ was coated with the polymer, where carboxyl groups were protected in form of ethyl ester. This coating was next unprotected by the basic hydrolysis. IR spectroscopy confirmed the change in the structure. Bands at 1750 and 1018 cm^−1^ at MNP-Ac spectrum (Supplementary Materials Figure S9) or 1732 and 1016 cm^−1^ at MNP-Mal (Supplementary Materials Figure S12) spectrum, characteristic for esters, disappeared after hydrolysis (Supplementary Materials Figures S10 and S13).

### 3.2. Adsorption of OGNs on the MNPs

The adsorption of DNA-20 at the surface of all three synthesized MNPs was tested. Co-polymeric coatings have a zwitterionic character; thus, IE and HI mode were investigated. In the first mode, the solution of analyte was mixed with water or 10 mM solution of NH_4_OAc at pH 3.5. 4.5, 5.5, or 6.5 before mixing with the conditioned adsorbent. After the OGN adsorption step, the supernatants were collected and analyzed. The detailed adsorption conditions and the obtained results are presented in Table 2.

Results shows that adsorption of OGN in IE mode strongly depends on co-polymer structure, pH, and the presence of salt. In the case of MNP-Ac, partial adsorption was possible from water solution without buffer addition or pH change. However, almost full adsorption was possible only when the analyte solution was mixed with buffer at pH < 4.5 (Table 2). Nearly full adsorption of DNA-20 on MNP-Sul and MNP-Mal was possible only on pH = 3.5. In other conditions, adsorption was impossible or significantly low (Table 2). In further studies, the adsorption of OGNs on MNP-Ac was performed at pH = 4.5, while, on MNP-Sul and MNP-Mal, adsorption was performed at pH = 3.5.

Investigated analytes are highly polar compounds with multiple negative charges. On the other hand, at the surface of the copolymer coated MNPs, there are positives charges on imidazolium rings and negative charges connected with the presence of carboxyl or sulphonate group. At low pH values, the negative charge on the surface of the adsorbent can be neutralized, and the negative charged OGNs can interact electrostatically with imidazolium rings. These interactions play a dominant role in analyte adsorption. Differences in adsorption effectiveness between three adsorbents can relate to different structures and differences in ligands protonation. As can be seen in the case of MNP-Ac, the addition of salt causes the increase of ionic strength of the solution and the lowering of the amount of adsorbed OGN. Considering the structures of OGNs and MNPs coatings, other possible interactions can be indicated, such as π interactions (between imidazolium rings and benzene rings of co-polymer and aromatic rings of nucleobases) or hydrogen bonding (between the carboxyl group and polar groups of nucleic acid bases).

For adsorption of OGN on MNPs in HI mode, the standard solution was mixed with an appropriate volume (50 or 450 µL) of organic solvent (ACN or ACE). Detailed conditions were presented in Table 2. Different tendencies were observed for various MNPs. For MNP-Ac, the retention does not depend on the type of organic solvent, but its volume. The degree of adsorption is higher when 50% *v*/*v* addition of organic solvent is applied. Almost 90% of DNA-20 was retained when the sample was mixed with 50 µL of ACN. Similarly, in the case of MNP-Sul organic solvent type does not influence adsorption efficiency. It depends on the volume of organic solvent, but contrary to MNP-Ac, the higher retention was possible when 90% *v*/*v* addition was applied. Unfortunately, the highest possible degree of adsorbed OGN was nearly 65%. In the case of MNP-Mal, the DNA-20 adsorption depends both on the types of organic solvent and its volume. The highest retention was possible when the addition of 450 µL of ACE was applied; it was equal to 80%.

Zwitterionic ligands of MNPs coatings strongly adsorb water by hydrogen bonding. In described conditions, when high organic solvent content is applied for adsorption, the water-rich layer on the adsorbent surface is created. The retention of polar analytes is predominantly caused by partition between this layer and the liquid phase with a higher content of organic solvent. Likewise, in the case of IE mode, other interactions are also possible.

**Figure 3:**
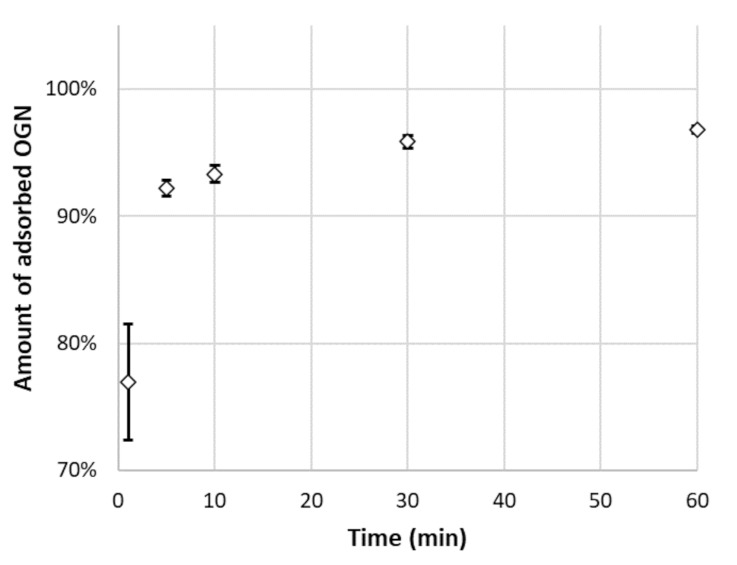
The impact of time on DNA-20 oligonucleotide adsorption effectiveness on MNP-Ac (oligonucleotide sequence is provided in Table 1, MNP-Ac adsorbent structure in Figure 2, and experimental conditions in Section 2.4). Abbreviations: OGN—oligonucleotide.

Among all tested modes and MNPs, the highest degree of adsorption was achieved for MNP-Ac, when the sample before the loading step was mixed with NH_4_OAc buffer at pH = 4.5. For these conditions, the impact of adsorption time on its effectiveness was investigated. The results of these investigations are presented in Figure 3. As can be seen, over 90% of OGN was retained on MNP-Ac after 5 min. Further extension of time only slightly increases the percentage of adsorbed DNA-20 (Figure 3). Relatively fast adsorption may relate to a strong electrostatic attraction between the positively charged polymer surface and negatively charged OGN molecules. The loading step time was appointed for 15 min, as a compromise between adsorption time and adsorption effectiveness.

The sorption capacity of MNP-Ac for DNA-20 was measured. The measurements were performed by following the procedure described in Section 2.4 for different sample weights (0.52, 0.77, and 1.00 mg). A 50 µL of 50 µM DNA-20 standard solution was mixed with the same volume of 10 mM NH_4_OAc (pH = 4.5), and then load into conditioned MPS1. After 15 min the supernatant was separated and analyzed. The process was carried out until OGN was detected in the supernatant. The adsorbent sorption capacity was calculated based on the amount of adsorbed OGN and was equal to 1.79 ± 0.16 µmol g^−1^ (RSD = 8.8%). This value allows for estimating the adsorbent mass required for the extraction of OGNs. Consequently, 2.0 mg of adsorbent was used during further studies.

### 3.3. Studies on OGN Desorption

The desorption of unmodified DNA-20 OGN from the three investigated MNPs in two modes was investigated.

#### 3.3.1. The IE Mode

**Figure 4:**
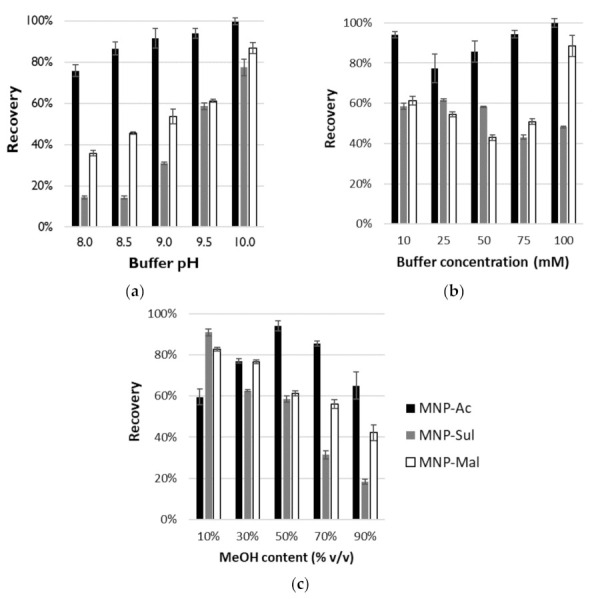
The influence of the elution solvent pH (**a**), and salt concentration (**b**), and methanol content (**c**) in the elution solvent on the extraction recovery for MNP-Ac (black bars), MNP-Sul (gray bars), and MNP-Mal (white bars). Experimental conditions in the text.

Adsorption of OGN on MNPs coated with crosslinked poly(ionic liquid)s on IE mode was performed at a low pH. For desorption, the solution of NH_4_OAc at appropriate pH in a mixture with MeOH was used. These starting conditions were taken from our previous investigations with zwitterionic poly(ionic liquid)s [22]. They showed, that the elution solvent pH, salt concentration, and methanol content in elution solvent have an impact on desorption efficiency. The influence of these three parameters on the extraction recovery of OGN from the MNPs was identified, are presented in Figure 4, and are described below.

The influence of the elution solvent pH on the extraction recovery: In the first step of desorption studies in IE mode, the impact of the elution solvent pH on DNA-20 recovery values was studied. The NH_4_OAc salt concentration was 10 mM, while MeOH content was 50% *v*/*v*. Other parameters were as described in Section 2.4. Five different pH values were tested: 8.0, 8.5, 9.0, 9.5, and 10.0. The results are plotted in Figure 4a. The pH of the elution solvent has a significant impact on the final recovery. The highest recovery (99.8 ± 1.7%) was obtained for MNP-Ac when the pH of the elution solution was equal to 10.0. At pH ≥ 9.0 the desorption of over 90% of DNA-20 was possible (Figure 4a). It was not possible to achieve such high recovery for two other MNP (Figure 4a). In both cases, the highest values of this parameter were obtained at pH = 10.0 and were equal to 77.4 ± 4.0% and 86.9 ± 2.8%, respectively, for MNP-Sul and MNP-Mal. Independently of which MNP was used, pH dependence on recovery was nearly linear for all MNPs. When pH increase, the values of recovery also increase (Figure 4a). Such an effect relates to the zwitterionic character of MNPs coating. During the adsorption of the OGN, at low pH values, the anionic groups of active ligands are protonated and electrostatic interaction between positively charged imidazolium rings and anionic analytes are possible. When pH increased, gradual deprotonation takes place, and electrostatic repulsion between negatively charged carboxyl or sulfonate groups and OGNs occurs. Thus, a more anionic analyte can be desorbed, and higher recovery values can be obtained.

The influence of salt concentration in the elution solvent on the extraction recovery. In the next step, the impact of the NH_4_OAc concentration on DNA-20 recovery values was investigated. The pH of the elution solution was equal to 9.5, the MeOH content was 50% *v*/*v*, and other parameters were as in the typical method (Section 2.4). In the studies, five different concentrations of salt were tested: 10, 25, 50, 75, and 100 mM. The results are shown in Figure 4b. The highest amount of OGN was desorbed from MNP-Ac and MNP-Mal using NH_4_OAc in the concentration equaled 100 mM. Obtained recoveries were 99.8 ± 2.2% and 88.6 ± 5.2% respectively. It is worth highlighting that, for MNP-Ac, the high values (~94%) were also obtained for concentrations 10 and 75 mM (Figure 4b). For MNP-Sul, the highest recovery was obtained for 25 mM concentration of salt in elution solution, and it was equal to 61.6 ± 0.6%. For all MNPs in the recovery dependence on NH_4_OAc concentration a similar trend is observed (Figure 4b). At the beginning, with the increase of concentration the recovery values decrease. At some NH_4_OAc concentrations, the desorption efficiency is the lowest. Above this point the recovery increase with an increase in the concentrations. The lowest values for each MNPs are obtained for different salt concentrations. They are 25, 75, and 50 mM for MNP-Ac, MNP-Sul, and MNP-Mal, respectively (Figure 4b). There is a certain analogy of the observed phenomenon to the effect described by Alpert for HILIC separations [27]. Probably, different mechanisms characterize interactions in the presence of different salt concentrations. Under the minimum recovery concentration, dissociated salt provides counterions for charged groups bounded to MNPs surface and makes electrostatic interaction of OGN with adsorbent easier. Above this point, desorption efficiency is proportional to salt concentration. This effect is characteristic for the ion-exchange mechanism and ion competition for electrostatic interaction with adsorbent active sites.

The influence of methanol content in the elution solvent on the extraction recovery. The influence of MeOH percentage in the elution solvent on the OGN desorption efficiency was also studied. The 10 mM solution of NH_4_OAc at pH = 9.5 was with MeOH mixed at appropriate volume ratio: 10, 30, 50, 70, and 90% *v*/*v*. Other extraction parameters were as described in Section 2.4. Obtained results were presented in Figure 4c. Appropriate MeOH content was significant for desorption efficiency. The highest recovery of DNA-20 from MNP-Ac was obtained for 50% *v*/*v* of MeOH in elution solvent. It is equal to 94.1 ± 2.4%. Both increasing and decreasing its content cause a decrease in the desorption efficiency (Figure 4c). An appropriate organic solvent addition allows for overcome of some additional forces causing the OGN retention, e.g., π–π bonds between aromatic rings of MNPs coating and heteroatom rings of nucleobases. Recovery losses in MeOH content above 50% *v*/*v* may relate to changes in the mechanism and increasing of participation of retention in high organic solvent content characteristic for HILIC separation. In opposite to MNP-Ac, this effect is dominant in the whole range of studied desorption conditions in the cases of two other MNPs: MNP-Sul and MNP-Mal (Figure 4c). The recoveries values decrease with an increase of MeOH percentage in elution solvent. The highest values were obtained for 10% *v*/*v* organic solvent addition and were equal to 90.9 ± 1.7% and 82.7 ± 1.0% for MNP-Sul and MNP-Mal, respectively (Figure 4c).

**Figure 5:**
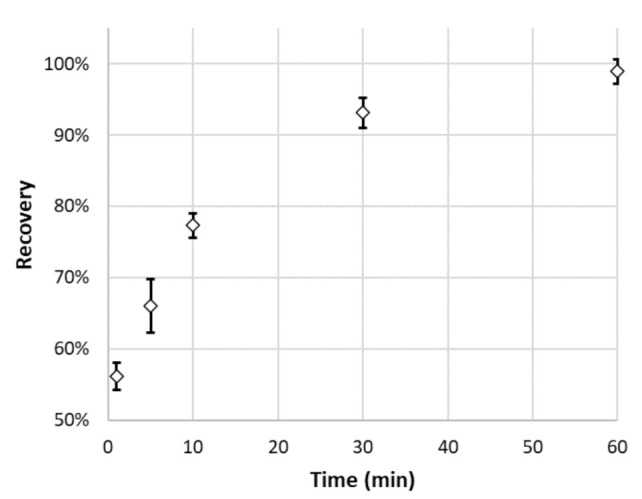
The impact of desorption time on DNA-20 oligonucleotide recovery from MNP-Ac (oligonucleotide sequence is provided in Table 1, MNP-Ac adsorbent structure on Figure 2, and experimental conditions in Section 2.4). Abbreviations: OGN—oligonucleotide.

The influence of desorption time on the extraction recovery. Finally, the impact of desorption time on its efficiency was studied. The desorption studies showed that the best results are obtained for MNP-Ac. Thus, this step of the research was performed only for this material. The desorption of DNA-20 was performed with 10 mM solution of NH_4_OAc at pH = 9.5 with addition of 50% *v*/*v* of MeOH at different times: 1, 5, 10, 30, and 60 min. Other extraction parameters were as described in Section 2.4. The obtained results are plotted in Figure 5. The largest amount of OGN was desorbed after 60 min. However, the recovery after 30 min was only ~6% percentage points lower, and it was still higher than 90%. Thus, the 30 min desorption time was applied in the final procedure as a compromise between good recovery and sample preparation time.

#### 3.3.2. The HI Mode

Partial adsorption of DNA-20 at the surface of investigated MNP was possible; thus, a study on the possibility of its desorption was performed. The OGN was loaded into adsorbent in conditions in which the adsorption degree was the highest (Table 3). Water has the highest elution strength on HILIC; thus, it was tested for elution of adsorbed OGN from MNP. Moreover, water alkalized with ammonia to pH = 9.5 was also investigated. Unfortunately, the recoveries in all cases were significantly low (Table 3). For MNP-Sul and MNP-Mal were below 20%. The highest desorption efficiency in HI mode was achieved for MNP-Ac and water ammonia solution at pH = 9.5 as an elution solution but is still less than 35%. As satisfactory results were not achieved, further studies on the HI mode extraction were abandoned.

### 3.4. Extraction of OGNs with Different Lengths and Modifications

Performed studies on the influence of parameters of adsorption and desorption of OGN on MNP allow for the determination of conditions for extraction to obtain the possible high recovery. The final MDSPE procedure is presented in Table 4. The MNP-Ac gives the highest recoveries, so this MNP was chosen for the final extraction procedure. The IE exchange mode was selected, and the adsorption was performed at pH = 4.5 (Table 4). In the elution step, the 10 mM solution of NH_4_OAc at pH = 9.5 in mixture with MeOH (50/50, *v*/*v*) was applied. Adsorption time was set at 15 min, and desorption time was set at 30 min (Table 4).

**Figure 6:**
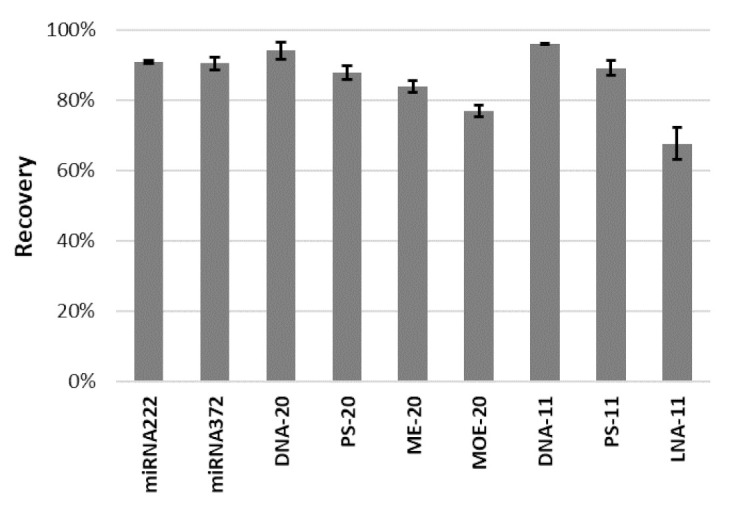
The recoveries obtained for the OGNs with different modification types and lengths in MDSPE with the use of MNP-Ac (extraction conditions in Table 4).

The final procedure (Table 4) was applied for oligonucleotides with a different sequence, length, and modifications. The sequences of 20-mer and 11-mer OGNs are analogical with Alicaforsen, the antisense OGN tested as a potential drug for pouchitis in enema formulation. The modifications investigated in the present studies are chosen from the most popular ones used in antisense drugs. Moreover, a microRNAs are potential diagnostic markers; thus, extraction of their analogs was also investigated. The recovery values obtained for each studied OGN are presented in Figure 6.

Except for MOE-20 and LNA-11, the recovery values in all cases are higher than 80%. Moreover, these values are higher than 90% for unmodified OGNs (Figure 6). Introducing a chemical modification in the structure of the OGN affects the hydrophobicity of the molecule. The phosphorothioate one, where the nonbinding oxygen atom in the phosphate group was replaced by a less electronegative sulfur atom, is more hydrophobic than an unmodified strand of DNA with the same length and sequence. Similarly, the substituent in the 2′ position of the sugar group influences this property. The longer the substituent, the more hydrophobic the OGN will be. The changes in this property can be a potential cause of changes in the recoveries values. While comparing results obtained for 20-mers, the highest values were obtained for unmodified OGN, they decreased with the decrease of hydrophobicity, and they are the lowest for the most hydrophobic MOE-20 (Figure 6). Similar tendencies can be seen for 11-mer OGNs. The lowest recovery was obtained for the LNA-11, the most hydrophobic in this group of analytes (Figure 6). It is worth highlighting that chemical modification of OGN can introduce additional interaction to the mechanism of retention of the analytes with MNP coating (e.g., C–H—π interactions). Nevertheless, the procedure, which was optimized for unmodified OGN, works in the case of OGNs with chemical modifications.

Based on the obtained results, some conclusions about the influence of OGN chain length on the recovery can be made. Comparing unmodified DNA and phosphorothioate OGN with 20 and 11 nucleotide length, the higher values were obtained for the shorter ones (Figure 6). They have a lower charge, and their interaction with charged MNP coating is weaker. Thus, their desorption can be easier.

### 3.5. Chromatographic Method Validation

Methods for quantification of OGNs are widely used during clinical studies, as well as in metabolism investigations. Thus, in our studies, we investigated the method for extraction and RP-UHPLC-DAD method for separation and determination of OGNs: DNA-16, DNA-18, and DNA-20. DNA-20 is a parent compound, while DNA-18 and DNA-16 are potential synthetic metabolites (shorter, with two or four nucleotides at the 3′ end).

**Figure 7:**
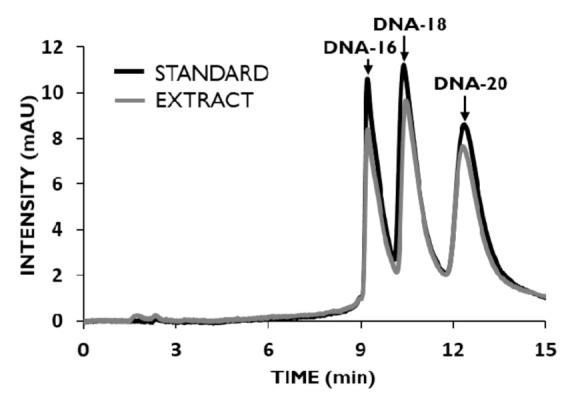
Chromatograms for the separation of DNA-20 and its synthetic metabolites DNA-18 and DNA-16 from standard solution (black line) and extract, obtained with developed MDSPE (gray line). Experimental conditions: mobile phase, 10 mM NH_4_OAc and MeOH; gradient elution, 5–20% of MeOH in 5 min and next 20–25% of MeOH in 10 min; chromatographic column ACE Excel C18, 1.7 μm; UV detection at λ = 260 nm; column and autosampler temperature, 30 °C; flow rate, 0.15 mL min^−1^; injection volume, 2 μL.

Based on earlier investigations performed in our group [28], separation of the OGNs mixture was performed by using an octadecyl UHPLC column and an aqueous solution of organic salt with MeOH addition as a mobile phase. Both ammonium acetate and ammonium formate were tested, in concentrations of 5, 10, and 25 mM (data not shown). The satisfactory separation was achieved by using a 10 mM solution of ammonium acetate in a gradient program in 15 min. Figure 7 presents an exemplary chromatogram for the separated mixture.

The extraction procedure that was developed during the present studies (Table 4) was tested for the extraction of parent OGN and its metabolites from standard solution. Recoveries obtained for components of the mixture at 5 µM concentration were equal to 89.8 ± 1.9%, 91.5 ± 1.6%, and 92.6 ± 1.9% for DNA-16, DNA-18, and DNA-20, respectively. These results showed that the method is suitable for usage in the extraction of OGN mixtures.

Finally, the developed extraction procedure was used as a sample preparation method for the OGNs mixture from fortified serum. Moreover, the samples were analyzed with the validated UHPLC–DAD method. Recovery and validation parameters for an analysis method (linearity, LOQ, LOD, and intraday and interday precision) were determined and are collected in Table 5. Intraday precision values were lower than 4.5% for low (1.25 µM), 3.6% for medium (7.5 µM), and 1.6% for high (15.0 µM) concentration. Repeatability did not exceed 7.5%. The LOD values were in the range of 0.28–0.32 µM (Table 5).

The recoveries values of OGNs from enriched serum samples were in the range of 82–85% (Table 5), wherein the matrix effect was in the range of 2.8–3.5%. As can be seen, the developed MDSPE procedure can be applied to real samples. It is important to note that the extraction of OGNs with the MNP coated with zwitterionic poly(ionic liquid) can be performed without any preliminary purification. This is the great advantage of the presented method, which confirms our previous studies on the application of poly(ionic liquid)s for OGNs extraction purposes [22]. Application of other commonly used adsorbents required an additional purification step to remove proteins, usually LLE or proteinase K digestion [9]. Moreover, the developed procedure was characterized by good recovery and repeatability.

## 4. Conclusions

The three MNPs coated with different crosslinked poly(ionic liquid)s were synthesized. They differed in functional ligands of bonded IL. All of them were imidazolium derivatives. Two of them have additional carboxyl groups, and the third one has a butyl sulfonate group. The polymers-coated MNP were characterized and used in further research on OGN extraction.

Adsorption of unmodified OGN on the MNP was possible at a low pH (ion-exchange mode) or with the addition of an appropriate volume of organic solvent (ACN or ACE—hydrophilic interaction mode).

Water and water alkalized with ammonia were tested for the desorption of OGNs adsorbed in HI mode. Unfortunately, obtained recovery values were low. Opposite results were obtained when the IE mode was applied. Regardless of MNP, desorption was possible with the use of alkaline water solutions of organic salt with the addition of methanol. However, the effects of salt concentration, pH, and organic solvent percentage were different for different MNP. The studies have shown that various interactions are responsible for OGNs adsorption; thus, a diverse desorption mechanism occurs. Based on the performed investigations, the MDSPE procedure for OGN extraction with the use of MNP coated with zwitterionic poly(ionic liquid) was proposed. This procedure uses 10 mM NH_4_OAc (pH = 9.5)/MeOH (50/50, *v*/*v*) as the elution solution.

The proposed procedure was used in the extraction of different tested analytes. They differed in lengths and chemical modifications. The obtained recoveries were higher than 80% for all unmodified DNA, RNA, phosphorothioate, and 2′-O-methyl OGNs. The desorption effectiveness was lower for molecules with less polar modifications, such as 2’-O-(2-methoxyethyl) and locked nucleic acid. In general, the extraction of shorter OGN gives higher recovery. This information is useful considering the potential applications.

Finally, the MDSPE procedure was tested in the serum sample preparation. The mixture of OGN and its synthetic metabolites was extracted from fortified serum with over 80% recoveries without any pretreatment or additional purification. Crosslinked poly(ionic liquid)s are promising adsorbents for extraction of OGNs. Coating magnetic nanoparticles with them greatly facilitates the extraction procedure when comparing with D-µ-SPE with pure adsorbents.

## Figures and Tables

**Table 1:** The oligonucleotides used in this study.

Abbreviation	Type of Modification	Molecular Mass (g mol^−1^)	Sequence 5′-3′
DNA-20	unmodified DNA	6063	GCCCAAGCTGGCATCCGTCA
DNA-18	unmodified DNA	5461	GCCCAAGCTGGCATCCGT
DNA-16	unmodified DNA	4827	GCCCAAGCTGGCATCC
DNA-11	unmodified DNA	3342	GCCCAAGCTGG
miRNA372	unmodified RNA	7609	AAAGUGCUGCGACAUUUGAGCGU
miRNA222	unmodified RNA	6910	AGCUACAUCUGGCUACUGGGU
PS-20	phosphorothioate	6368	GCCCAAGCTGGCATCCGTCA
PS-11	phosphorothioate	3503	GCCCAAGCTGG
ME-20	2’-O-methyl	6621	GCCCAAGCTGGCATCCGTCA
MOE-20	2’-O-(2-methoxyethyl)	7657	GCCCAAGCTGGCATCCGTCA
LNA-11	locked nucleic acid	3706	GCCCAAGCTGG

**Table 2:** Amounts of DNA-20 adsorbed on tested MNPs in different modes. Abbreviations: OGN—oligonucleotide; ACN—acetonitrile; ACE—acetone.

Extraction Mode	Conditions	Amount of Adsorbed OGN		
		MNP-Ac	MNP-Sul	MNP-Mal
Ion-exchange	50 µL DNA-20 5 µM + 50 µL H_2_O	63.2 ± 3.5%	0	0
	50 µL DNA-20 5 µM + 50 µL NH_4_OAc pH = 6.5	36.5 ± 1.3%	0	0
	50 µL DNA-20 5 µM + 50 µL NH_4_OAc pH = 5.5	33.8 ± 1.0%	0	0
	50 µL DNA-20 5 µM + 50 µL NH_4_OAc pH = 4.5	94.5 ± 3.8%	0	27.2 ± 3.6%
	50 µL DNA-20 5 µM + 50 µL NH_4_OAc pH = 3.5	93.3 ± 2.1%	93.1 ± 0.8%	93.1 ± 2.3%
Hydrophilic interactions	50 µL DNA-20 5 µM + 50 µL ACN	89.3 ± 2.3%	45.1 ± 1.3%	68.9 ± 2.1%
	50 µL DNA-20 5 µM + 450 µL ACN	59.5 ± 3.4%	64.1 ± 2.7%	50,5 ± 1.9%
	50 µL DNA-20 5 µM + 50 µL ACE	83.6 ± 0.7%	43.3 ± 1.7%	37.4 ± 2.5%
	50 µL DNA-20 5 µM + 450 µL ACE	65.2 ± 1.5%	62.7 ± 1.1%	80.1 ± 2.9%

**Table 3:** The conditions used for the extraction of DNA-20 in the HI mode. Abbreviations: MNP—magnetic nanoparticles; ACN—acetonitrile; ACE—acetone.

MNP	Conditioning	Adsorption	Desorption	Recovery
MNP-Ac	50 µL H_2_O/ACN 50/50 *v*/*v*	50 µL DNA20 5 µM + 50 µL ACN	50 µL H_2_O	23.4 ± 0.6%
			50 µL NH_3(aq)_ pH = 9.5	34.7 ± 1.1%
MNP-Sul	50 µL H_2_O/ACN 50/450 *v*/*v*	50 µL DNA20 5 µM + 450 µL ACN	50 µL H_2_O	13.2 ± 0.3%
			50 µL NH_3(aq)_ pH = 9.5	16.9 ± 0.3%
MNP-Mal	50 µL H_2_O/ACE 50/450 *v*/*v*	50 µL DNA20 5 µM + 450 µL ACE	50 µL H_2_O	13.7 ± 0.5%
			50 µL NH_3(aq)_ pH = 9.5	16.8 ± 0.2%

**Table 4:** The final procedure used for the MDSPE of modified and unmodified OGNs. Abbreviations: MeOH—methanol; NH_4_OAc—ammonium acetate.

Adsorbent	2.0 mg of MNP-Ac
Conditioning	100 µL MeOH, mixing, magnetic separation, supernatant remove
	100 µL 10 mM NH_4_OAc pH = 4.5, mixing, magnetic separation, supernatant remove
Sample load	50 µL sample + 50 µL 10 mM NH_4_OAc pH = 4.5, 15 min of mixing, magnetic separation, supernatant remove
Washing	100 µL 10 mM NH_4_OAc pH = 4.5, mixing, magnetic separation, supernatant remove
Elution	50 µL 10 mM NH_4_OAc pH = 9.5/MeOH 50/50 *v*/*v*, 30 min of mixing, magnetic separation, supernatant remove, centrifugation (14,000 rpm, 10 min), analysis

**Table 5:** Calibration curve parameters and validation parameters for determined OGNs. Abbreviations: OGN—oligonucleotide; R^2^—coefficient of determination; LOD—limit of detection; LOQ—limit of quantification; RSD—relative standard deviation.

OGN		DNA-16	DNA-18	DNA-20
Concentration range		1.25–20.0 μM		
Calibration curve equation		y = 1.3675x − 1.2946	y = 2.0206x − 1.6838	y = 1.8008x − 1.6348
R^2^		0.9996	0.9996	0.9995
LOD (µM)		0.29	0.28	0.32
LOQ (µM)		0.95	0.92	1.08
RSD (%) intra-day	1.25 µM	2.90	3.59	4.93
	7.5 µM	2.12	2.40	3.59
	15.0 µM	1.52	1.60	1.49
RSD (%) inter-day	1.25 µM	7.45	6.67	7.26
	7.5 µM	5.55	5.68	4.95
	15.0 µM	3.75	3.54	3.18
Recovery (%)		82.1 ± 2.1	82.5 ± 3.3	85.0 ± 3.6

## Data Availability

The data presented in this study are available in article and Supplementary Materials.

## Supplementary Materials

The following are available online at https://www.mdpi.com/article/10.3390/ma14123146/s1, schemes and detailed synthesis procedures, IR and ^1^H NMR spectra, SEM images (Supplementary Materials Figures S1–S14).
