# Supplementary material for: Application of Magnetic Nanoparticles Coated with Crosslinked Zwitterionic Poly(ionic liquid)s for the Extraction of Oligonucleotides
Source: Materials (Basel). 2021 Jun 8;14(12):3146. doi: 10.3390/ma14123146 (PMC8226603; doi:10.3390/ma14123146)
Supplement: Supplementary file 1 [file materials-14-03146-s001.zip › materials-1246574-SM-update.pdf]

materials

MDPI

Supplementary Materials

Application of Magnetic Nanoparticles Coated with Crosslinked Zwitterionic Poly(ionic liquid)s for the Extraction of Oligonucleotides

Łukasz Nuckowski [1,*], Krzysztof Dzieszkowski [2], Zbigniew Rafiński [2] and Sylwia Studzińska [1]

1 Chair of Environmental Chemistry and Bioanalytics, Faculty of Chemistry, Nicolaus Copernicus University in Toruń, 7 Gagarin Str., PL-87-100 Toruń, Poland; kowalska@chem.umk.pl
2 Chair of Organic Chemistry, Faculty of Chemistry, Nicolaus Copernicus University in Toruń, 7 Gagarin Str., PL-87-100 Toruń, Poland; dzieszko@doktorant.umk.pl (K.D.); payudo@chem.umk.pl (Z.R.)
* Correspondence: l.nuc@doktorant.umk.pl; Tel.: +48-56-611-4308; Fax: +48-56-611-4837

Abstract: Magnetic nanoparticles coated with zwitterionic poly(ionic liquid)s were applied for dispersive solid-phase extraction of oligonucleotides. The materials were synthesized by miniemulsion co-polymerization of ionic liquids and divinylbenzene on magnetic nanoparticles. The functional monomers contain a positively charged imidazolium ring and one of the anionic groups: derivatives of acetate, malonate, or butyl sulfonate ions. Adsorption of unmodified DNA oligonucleotide on obtained materials was possible in ion-exchange (IE) and hydrophilic interactions (HI) mode. The adsorption in IE was possible at low pH and was almost complete. The adsorption in HI mode required the usage of appropriate addition of organic solvent but did not provide full adsorption. Studies on the desorption of the analytes included determining the impact of ammonium acetate concentration and pH and organic solvents addition on the recovery. The material containing acetic fragments as an anionic group was selected for the final procedure with the use of 10 mM ammonium acetate (pH = 9.5)/methanol (50/50, v/v) as an elution solution. The magnetic dispersive solid-phase extraction procedure was tested for the oligonucleotides with various modifications and lengths. Moreover, it was applied to extract DNA oligonucleotide and its synthetic metabolites from enriched human plasma without any pre-purification with recoveries greater than 80%.

Keywords: antisense oligonucleotides; magnetic nanoparticles; poly(ionic liquid)s; magnetic dispersive sol-id-phase extraction; serum samples

Citation: Nuckowski, Ł.; Dzieszkowski, K.; Rafiński, Z.; Studzińska, S. Application of Magnetic Nanoparticles Coated with Crosslinked Zwitterionic Poly(ionic liquid)s for the Extraction of Oligonucleotides. *Materials* **2021**, *14*, 3146. https://doi.org/10.3390/ma14123146

Academic Editor(s): Gennady L. Gutsev

Received: 19 May 2021
Accepted: 5 June 2021
Published: 8 June 2021

Publisher's Note: MDPI stays neutral with regard to jurisdictional claims in published maps and institutional affiliations.

Copyright: © 2021 by the authors. Submitted for possible open access publication under the terms and conditions of the Creative Commons Attribution (CC BY) license (http://creativecommons.org/licenses/by/4.0/).

1. Synthesis of polymerizable IL and MNPs

1.1. The 3-(2-etoxy-2-oxoethyl)-1-vinylimidazolium bromide (EtAcviimBr)

Chloroform (10 mL), *N*-vinylimidazole (0.91 mL; 10 mmol), and ethyl bromoacetate (1.34 mL; 12 mmol) were added to a 25 mL round-bottom flask. The mixture was refluxed for 24 h with stirring under an argon atmosphere. Chloroform was removed using a rotary evaporator. The obtained white powder was washed three times with freshly distilled diethyl ether and dried under vacuum. Yield: 98.2%. ^{1}H NMR (700 MHz, $CDCl_3$) δ ppm: 1.33 (t, J=7.10 Hz, 3 H), 4.30 (q, J=7.10 Hz, 2 H), 5.49 (dd, J=8.50, 3.12 Hz, 1 H), 5.54 (s, 2 H), 5.92 (dd, J=15.49, 3.23 Hz, 1 H), 7.29 (dd, J=15.70, 8.60 Hz, 1 H), 7.52 (d, J=13.55 Hz, 2 H), 11.03 (s, 1 H). FT-IR (ATR, ν, cm^{-1}): 3453, 3143, 3122, 3100, 3084, 2992, 2966, 2932, 2820, 1748, 1657, 1645, 1574, 1548, 1429, 1392, 1372, 1349, 1221, 1181, 1097, 1018, 973, 940, 919, 882, 864, 785, 762, 701, 627, 573.

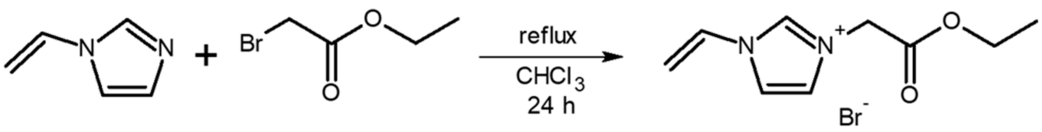

Scheme S1. Synthesis of EtAcviimBr.

1.2. The 4-(1-vinylimidazoliumyl)-butylsulphate (Sulviim)

Toluene (10 mL), *N*-vinylimidazole (0.91 mL; 10 mmol), and 1,4-butane sultone (1.53 mL; 15 mmol) were added to a 25 mL round-bottom flask. The mixture was heated in 70 °C for 24 h with stirring under an argon atmosphere. Obtained suspension was filtered, washed with toluene, pentane, and dried under vacuum. A white powder was obtained. Yield: 20.0%. ^{1}H NMR (700 MHz, D_2O) δ ppm: 1.66 (dtd, J=9.60, 7.80, 7.80, 5.90 Hz, 2 H), 1.95 (dt, J=15.17, 7.37 Hz, 2 H), 2.85 (m, J=7.60, 7.60 Hz, 2 H), 4.19 (t, J=7.10 Hz, 1 H), 5.31 (dd, J=8.66, 2.85 Hz, 1 H), 5.69 (dd, J=15.60, 2.80 Hz, 1 H), 7.03 (dd, J=8.70, 0.40 Hz, 1 H), 7.49 - 7.50 (m, 1 H), 7.66 - 7.68 (m, 1 H), 8.95 - 8.97 (m, 1 H). FT-IR (ATR, ν, cm^{-1}): 3143, 3104, 3080, 2936, 2919, 2861, 1663, 1646, 1584, 1552, 1469, 1441, 1421, 1377, 1332, 1291, 1262, 1190, 1175, 1123, 1064, 1036, 961, 930, 863, 787, 779, 742, 651, 596, 543, 520, 505, 448, 406.

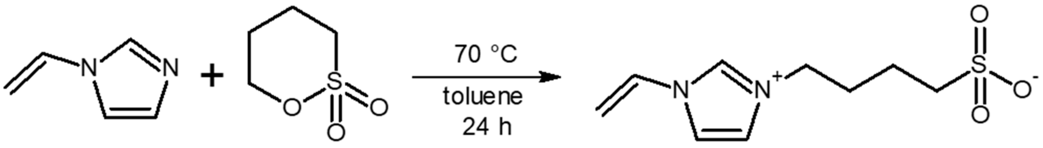

Scheme S2. Synthesis of Sulviim.

1.3. The 4-(1-vinylimidazoliumyl)-butylsulphate (Sulviim)

The EtMalviimBr was synthesized according to the procedure for EtAcviimBr, with the usage of diethyl bromomalonate (2.04 mL; 12 mmol) instead of ethyl bromoacetate. A red powder was obtained. Yield: 92.1%. ^{1}H NMR (700 MHz, $CDCl_3$) δ ppm: 1.34 (t, J=7.10 Hz, 6 H), 4.31 (dq, J=10.80, 7.10 Hz, 2 H), 4.38 (dq, J=10.80, 7.10 Hz, 2 H), 5.50 (dd, J=8.60, 3.44 Hz, 1 H), 5.94 (dd, J=15.49, 3.23 Hz, 1 H), 7.25 (s, 1 H), 7.33 (dd, J=15.60, 8.71 Hz, 1 H), 7.62 (s, 1 H), 7.89 (t, J=1.83 Hz, 1 H), 11.42 (s, 1 H). FT-IR (ATR, ν, cm^{-1}): 3125, 3058, 2973, 2833, 1759, 1736, 1669, 1567, 1557, 1466, 1376, 1305, 1282, 1248, 1221, 1191, 1161, 1097, 1059, 1007, 953, 942, 889, 857, 793, 775, 741, 645, 635, 603.

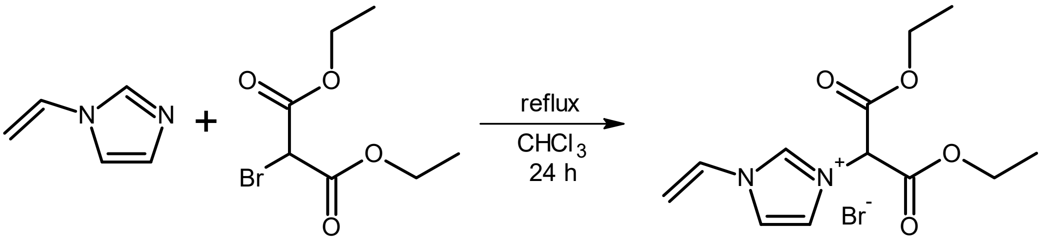

Scheme S3. Synthesis of EtMalviimBr.

1.4. Magnetic nanoparticles (Fe_3O_4)

A solution of 3.03 g $FeCl_3 \times 6H_2O$ (11.2 mmol) and 1.56 g $FeSO_4 \times 7H_2O$ (5.6 mmol) in 150 mL of water was heated to 50 °C under vigorous stirring and deaerated by bubbling with argon. Next, 12.5 mL of concentrated ammonia solution was added. After 30 min, the precipitate was collected by a magnet and washed three times with deionized water and methanol. Then, obtained black powder was dried under a vacuum.

1.5. MPS modified magnetic nanoparticles (Fe_3O_4-MPS)

1 g of dried Fe_3O_4 were ultrasonically dispersed in 100 mL of ethanol/water mixture (1:1, v/v). Next, 1 mL of MPS was added dropwise. The suspension was heated at 60 °C and vigorously stirred for 6 h. The product was collected by a magnet, washed three times with deionized water, ethanol, and dried under vacuum.

1.6. Magnetic nanoparticles coated with poly(2-(1-vinylimidazoliumyl)acetate-co-divinylbenzene) (MNP-Ac)

MNP coating: Fe_3O_4-MPS (0.35 g) was dispersed with the assistance of ultrasound in a mixture of 0.72 mL of DVB, 1.306 g of EtAcviimBr, and 1 mL of chloroform to form the oil phase. A solution of 0.065 g of SDS, 0.044 g of $Na_2HPO_4 \times 12H_2O$, and 0.017 g of NaH_2PO_4 in 25 mL of water was adjusted to pH 7 to form the water phase. The oil and the water phases were combined and sonicated to form a miniemulsion. Then, the mixture was placed in a 100 mL three-neck flask equipped with a condenser and mechanical stirrer and heated to 70 °C. Next, 0.015 g $K_2S_2O_8$ dissolved in 1 mL of water was added. The polymerization was performed under an argon atmosphere for 24 h. The obtained particles were separated by a strong magnet, washed three times with water, methanol, and dried under a vacuum.

MNP deprotection: the dry particles (0.75 g) were dispersed with the use of ultrasounds in the solution of 0.4 g of NaOH in the mixture of 9 mL of water and 1 mL of methanol. The mixture was placed in a 25 mL round-bottom flask and refluxed with mixing for 6 h. The obtained MNP-Ac were washed with 1 M HCl, three times with water and methanol, and dried under vacuum.

1.7. Magnetic nanoparticles coated with poly(4-(1-vinylimidazoliumyl)-butylsulphate-co-divinylbenzene) (MNP-Sul)

The MNP-Sul was prepared analogously to the procedure of MNP coating for MNP-Ac, with the usage of Sulviim (1.15 g) instead of EtAcviimBr. The obtained MNP-Ac were washed with 1 M HCl, three times with water and methanol, and dried under vacuum.

1.8. Magnetic nanoparticles coated with poly(2-(1-vinylimidazoliumyl)propanedioate-co-divinylbenzene) (MNP-Mal)

The MNP-Mal was prepared analogously to the procedure for MNP-Ac, with the usage of EtMalviimBr (1.665 g) instead of EtAcviimBr.

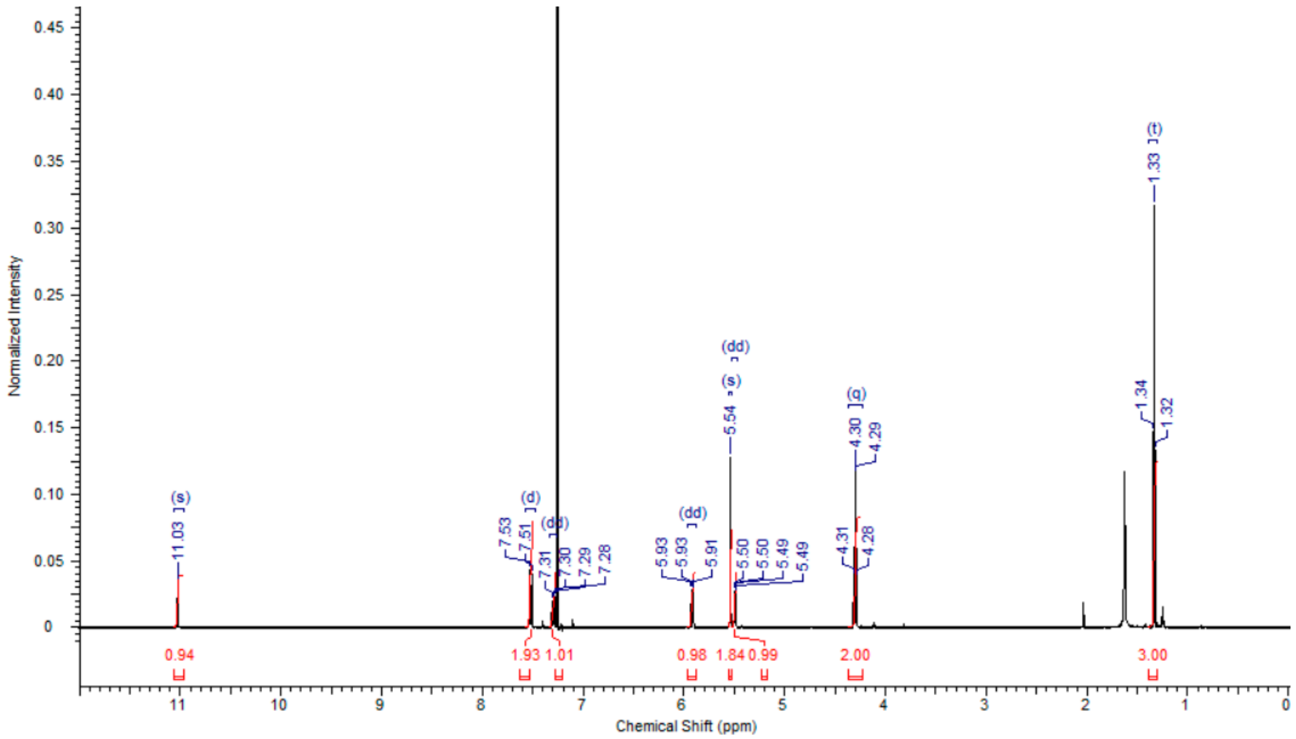

Figure S1. ¹H NMR spectrum EtAcviimBr.

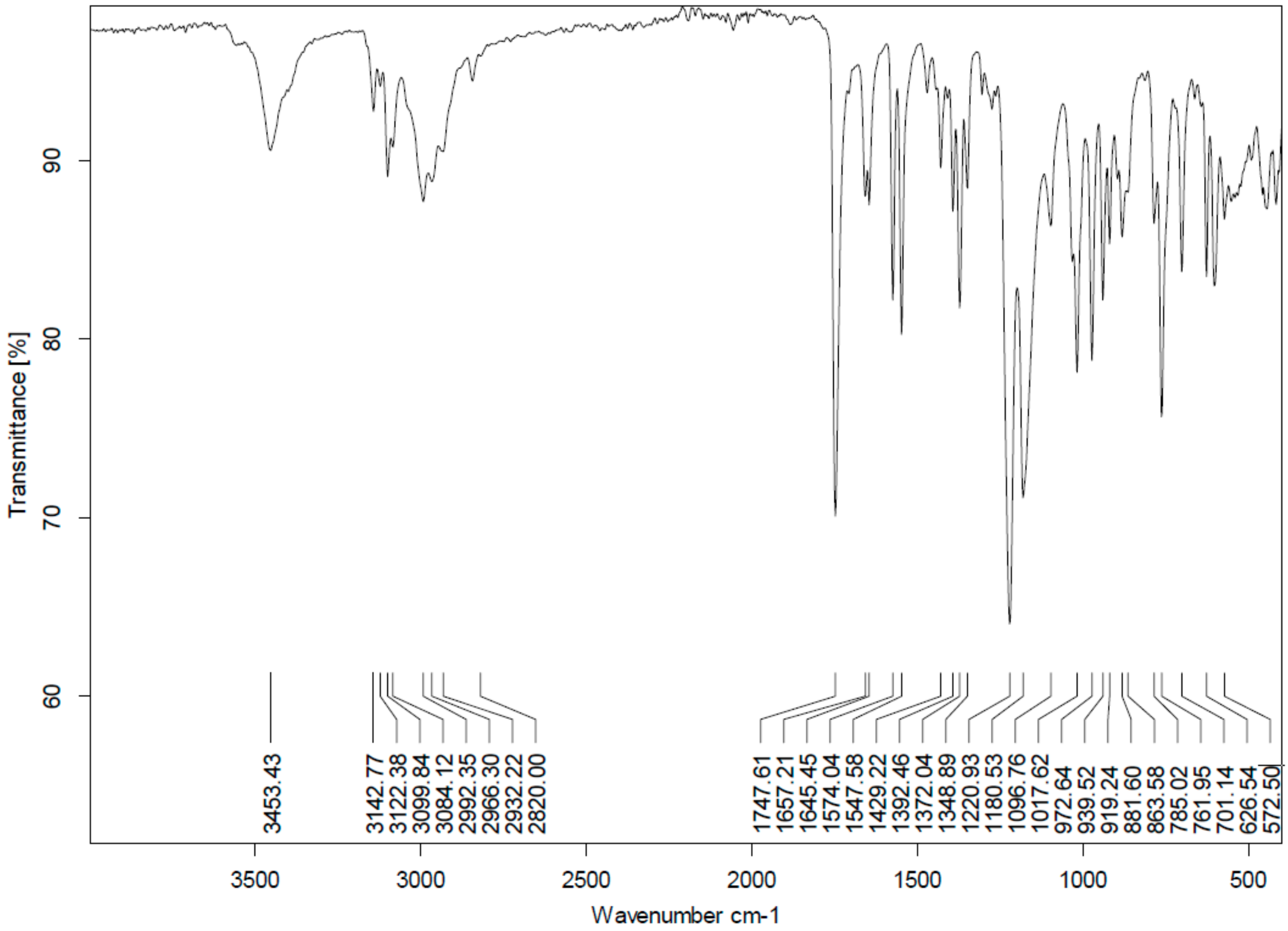

Figure S2. FT-IR spectrum of EtAcviimBr.

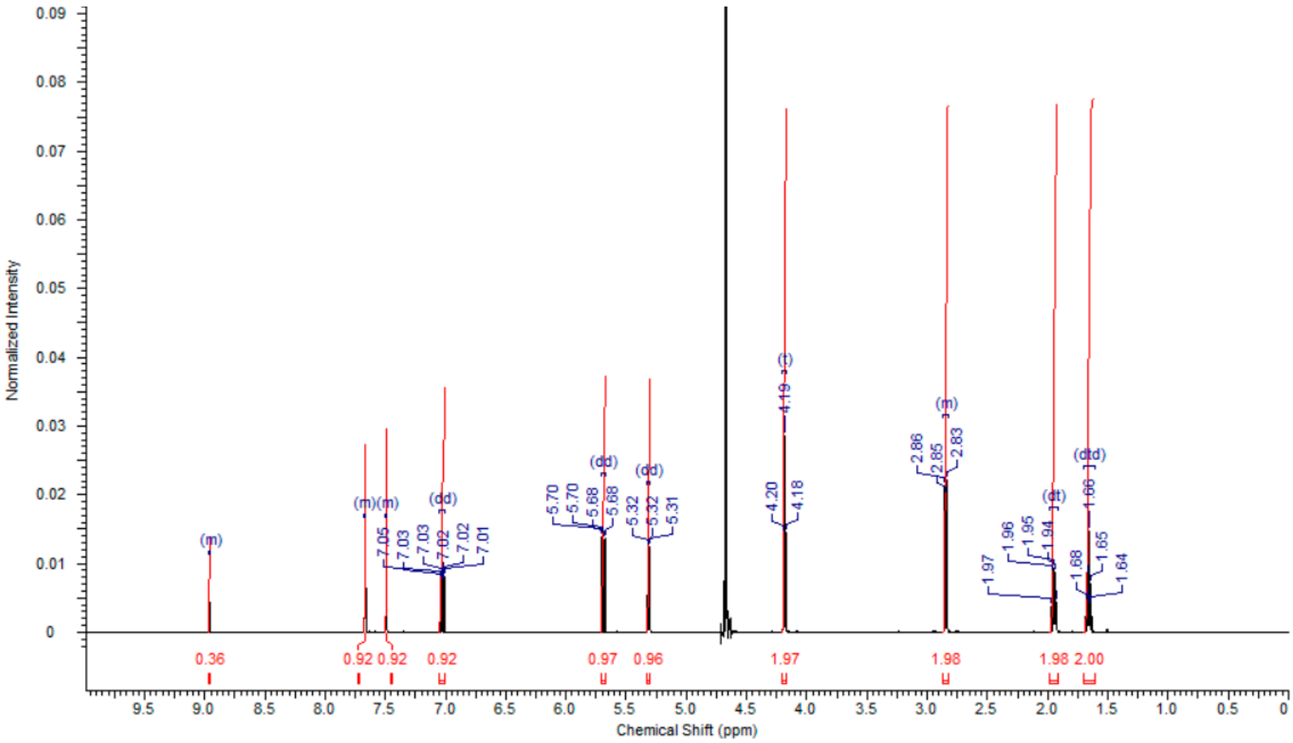

Figure S3. ^{1}H NMR spectrum of Sulviim.

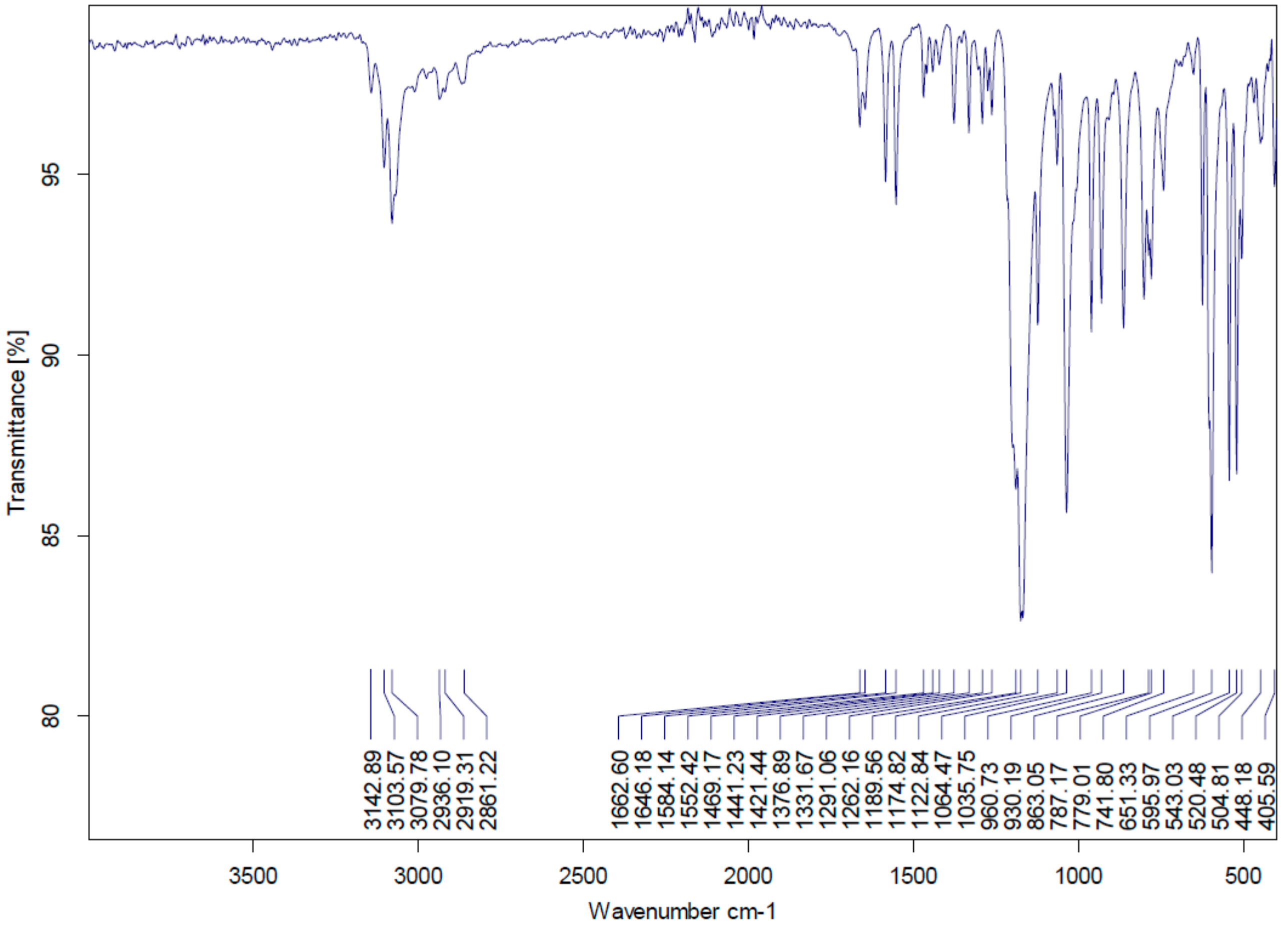

Figure S4. FT-IR spectrum of Sulviim.

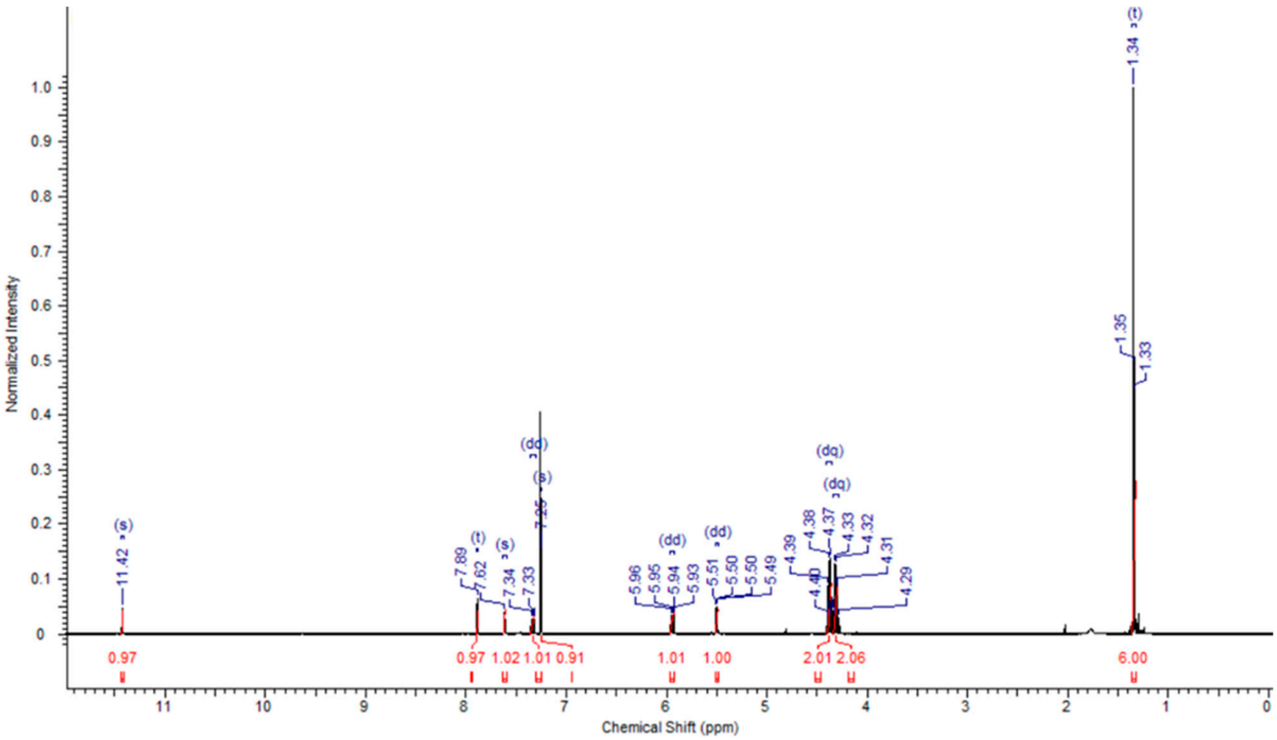

Figure S5. ^{1}H NMR spectrum of EtMalviimBr.

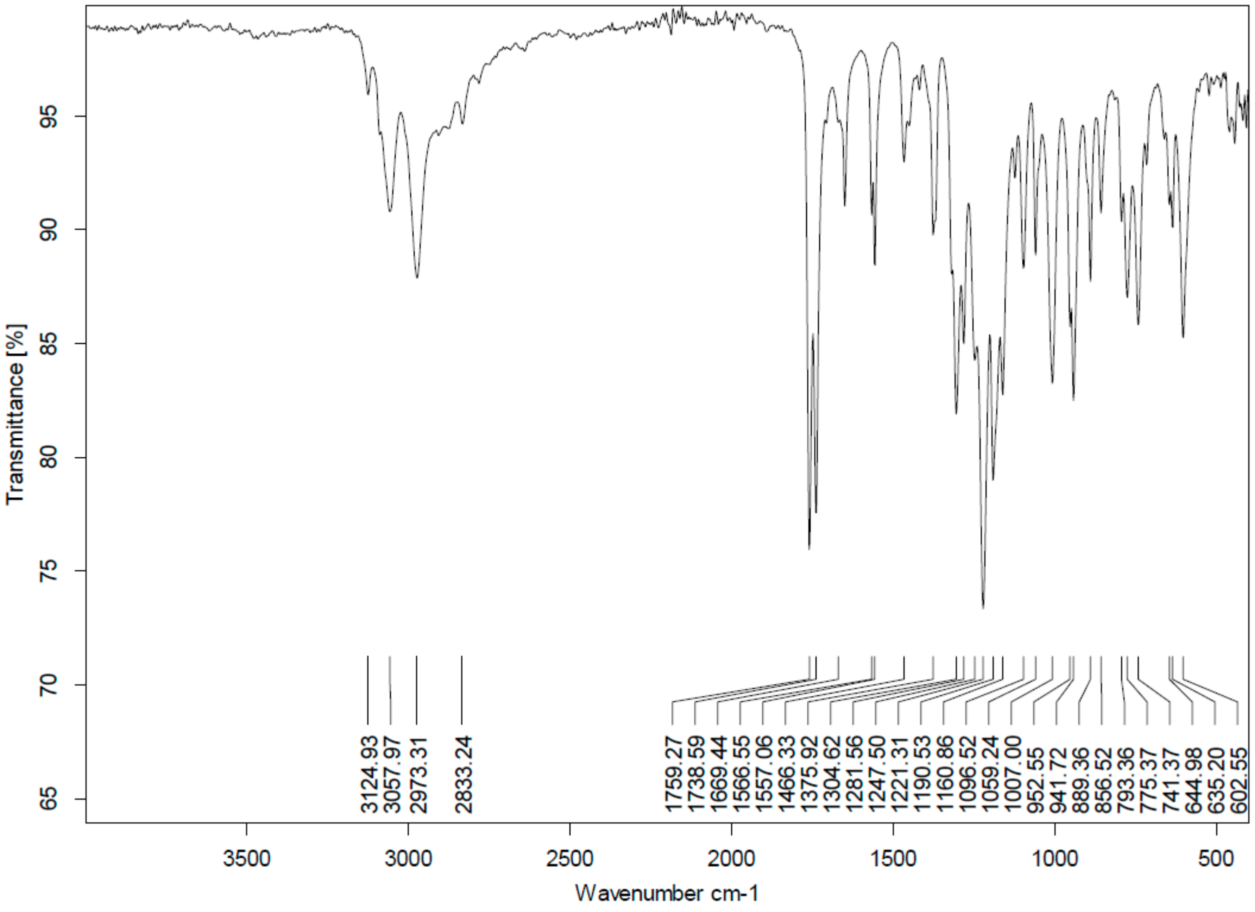

Figure S6. FT-IR spectrum of EtMalviimBr.

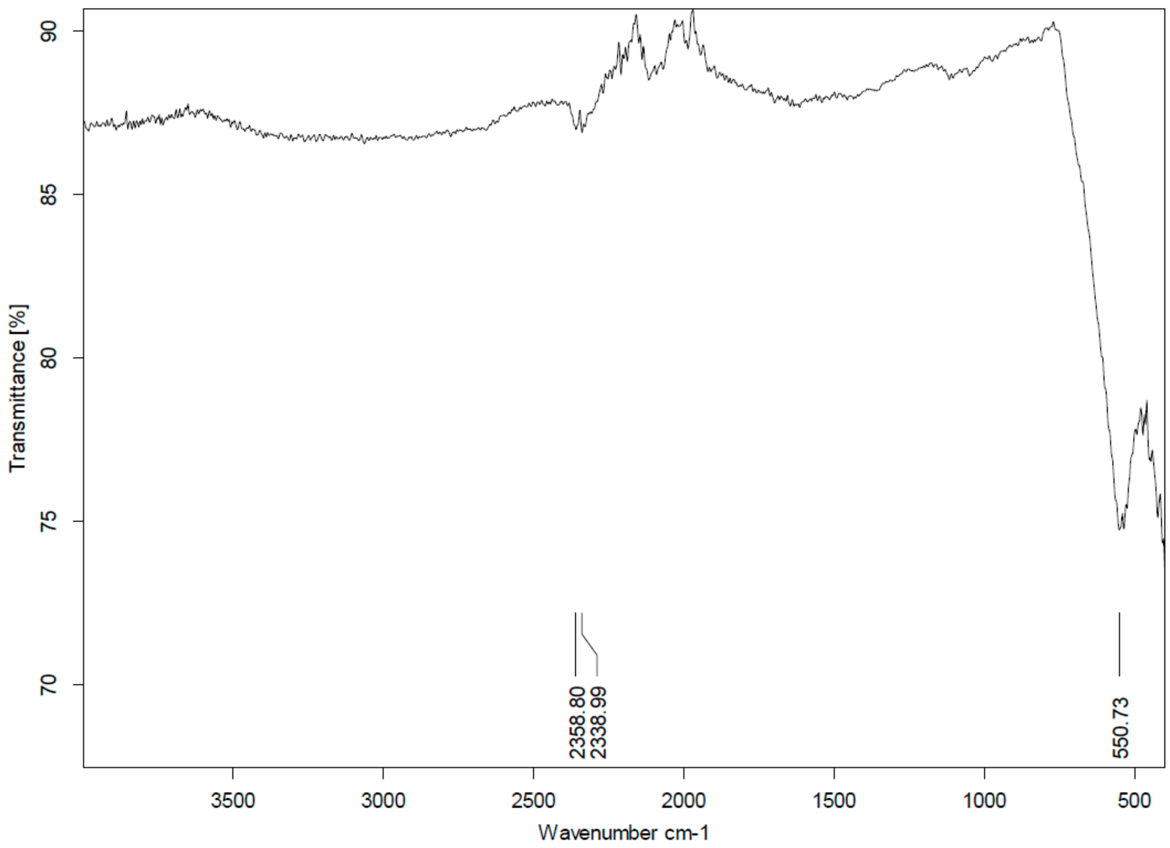

Figure S7. FT-IR spectrum of Fe_3O_4.

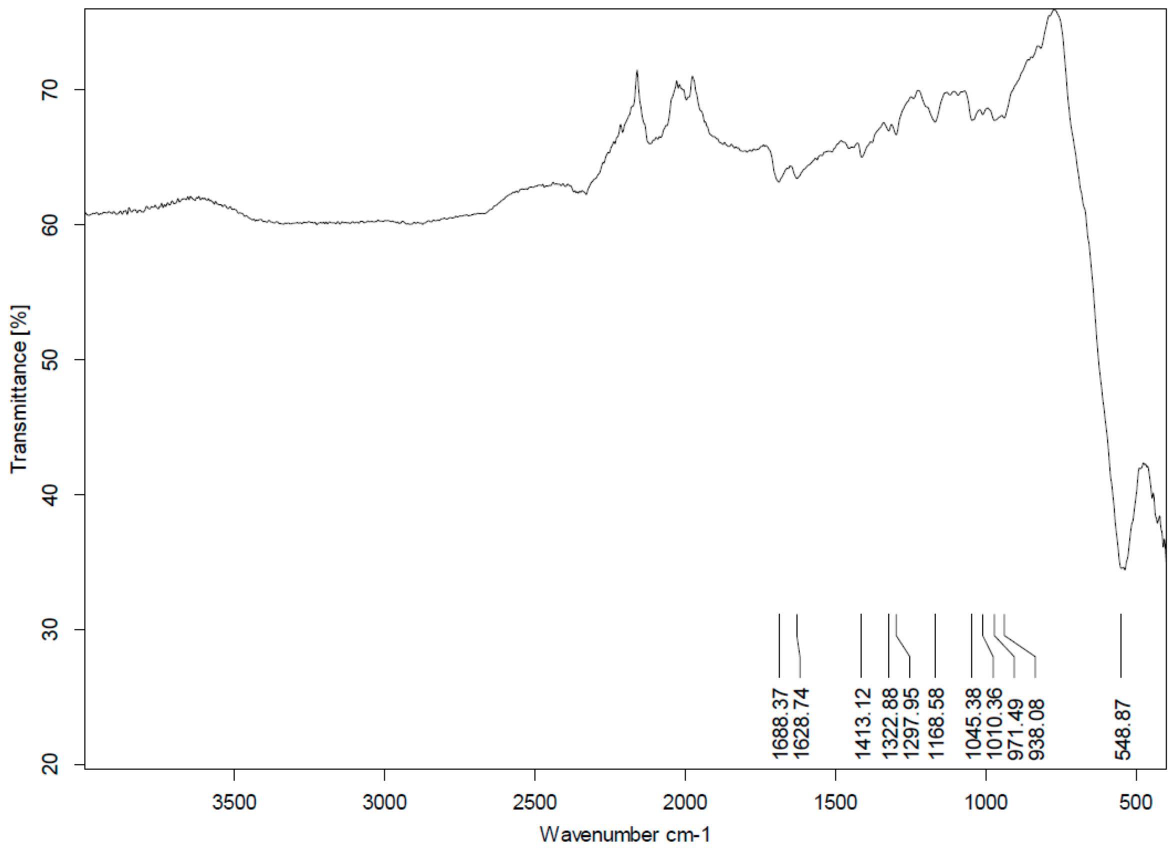

Figure S8. FT-IR spectrum of Fe_3O_4-MPS.

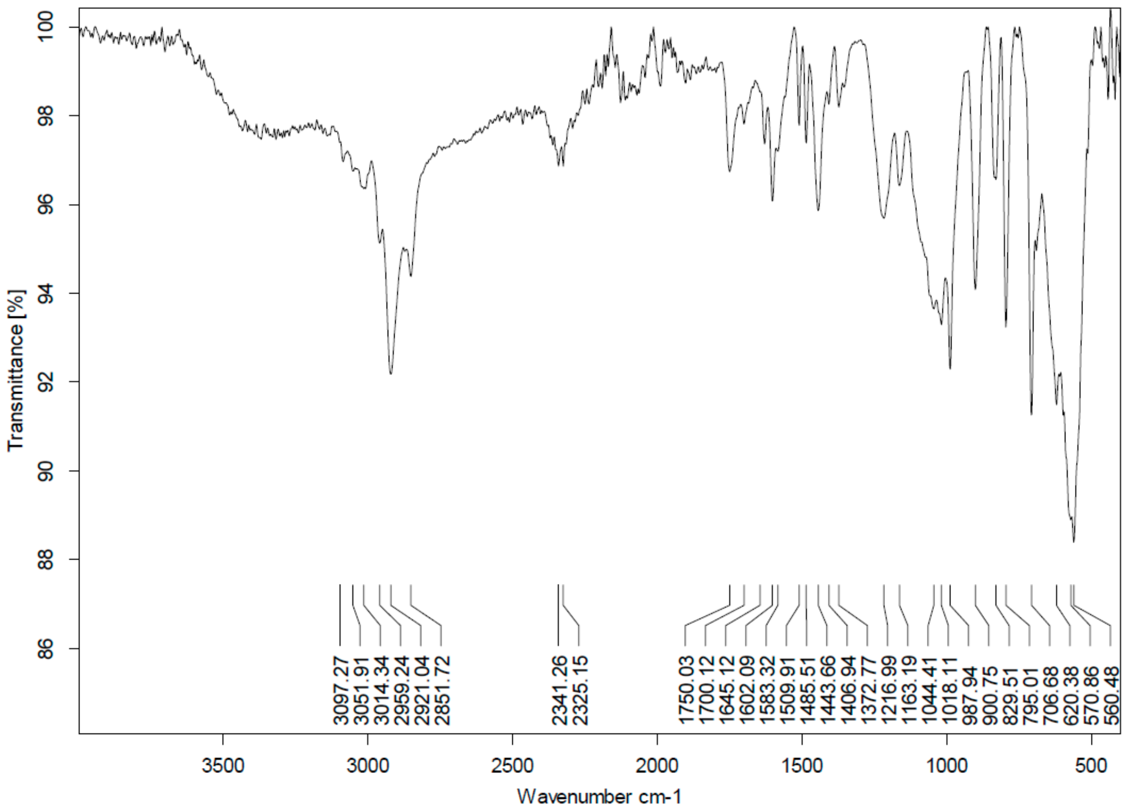

Figure S9. FT-IR spectrum of MNP-Ac before hydrolysis.

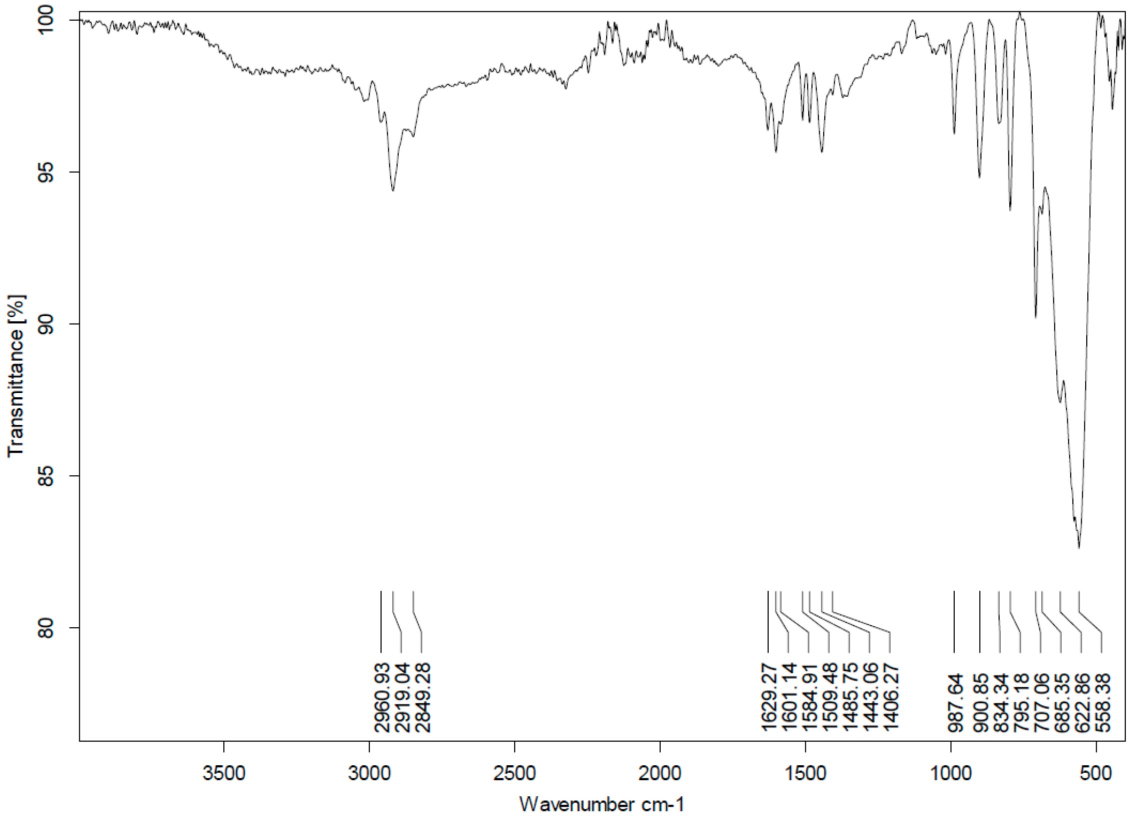

Figure S10. FT-IR spectrum of MNP-Ac after hydrolysis.

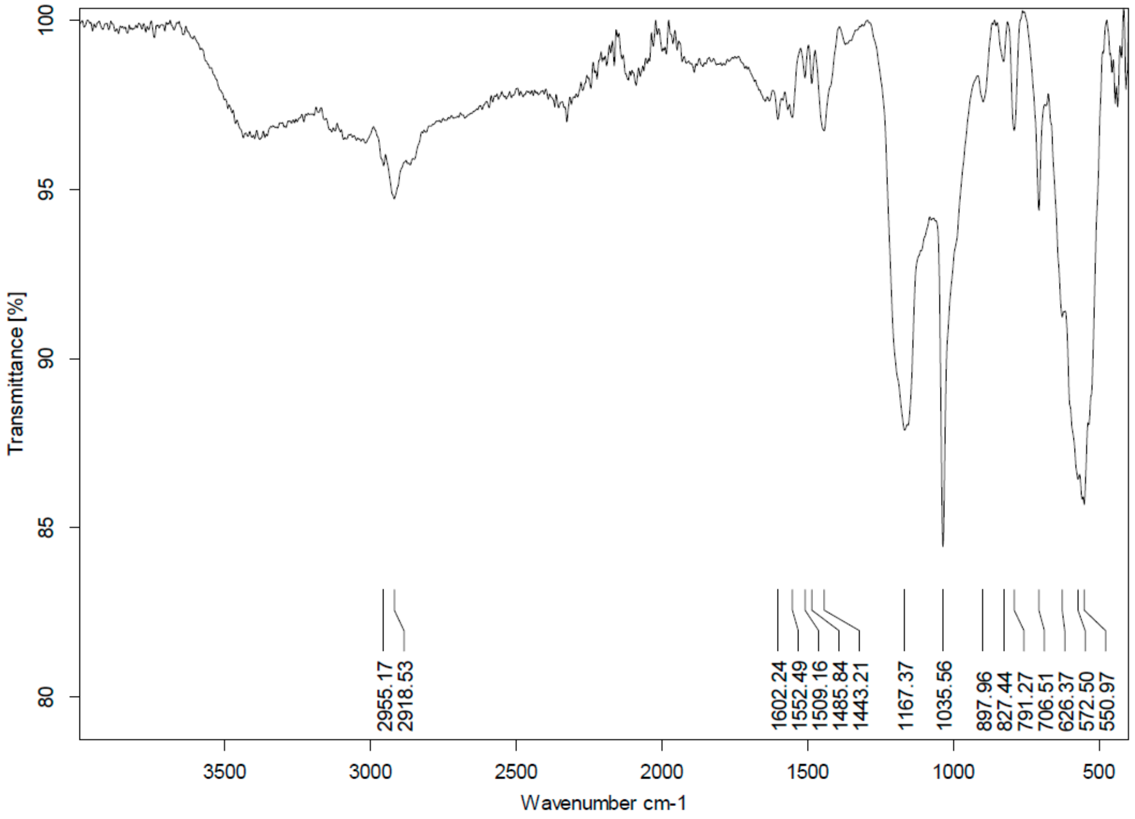

Figure S11. FT-IR spectrum of MNP-Sul.

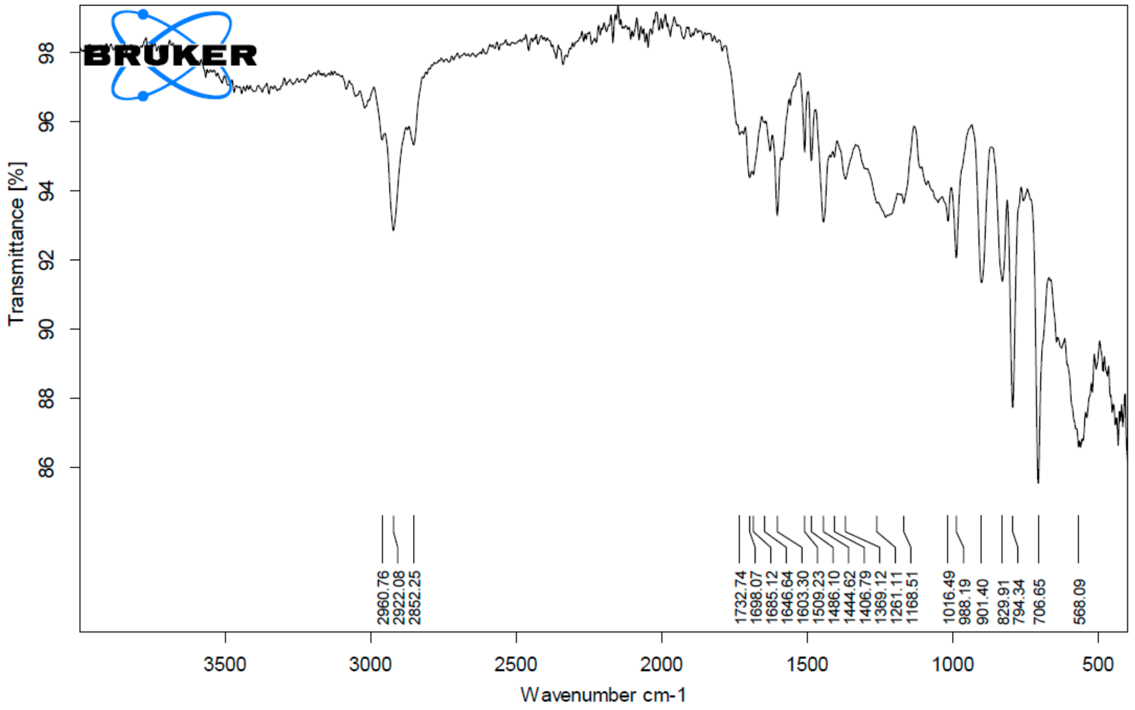

Figure S12. FT-IR spectrum of MNP-Mal before hydrolysis.

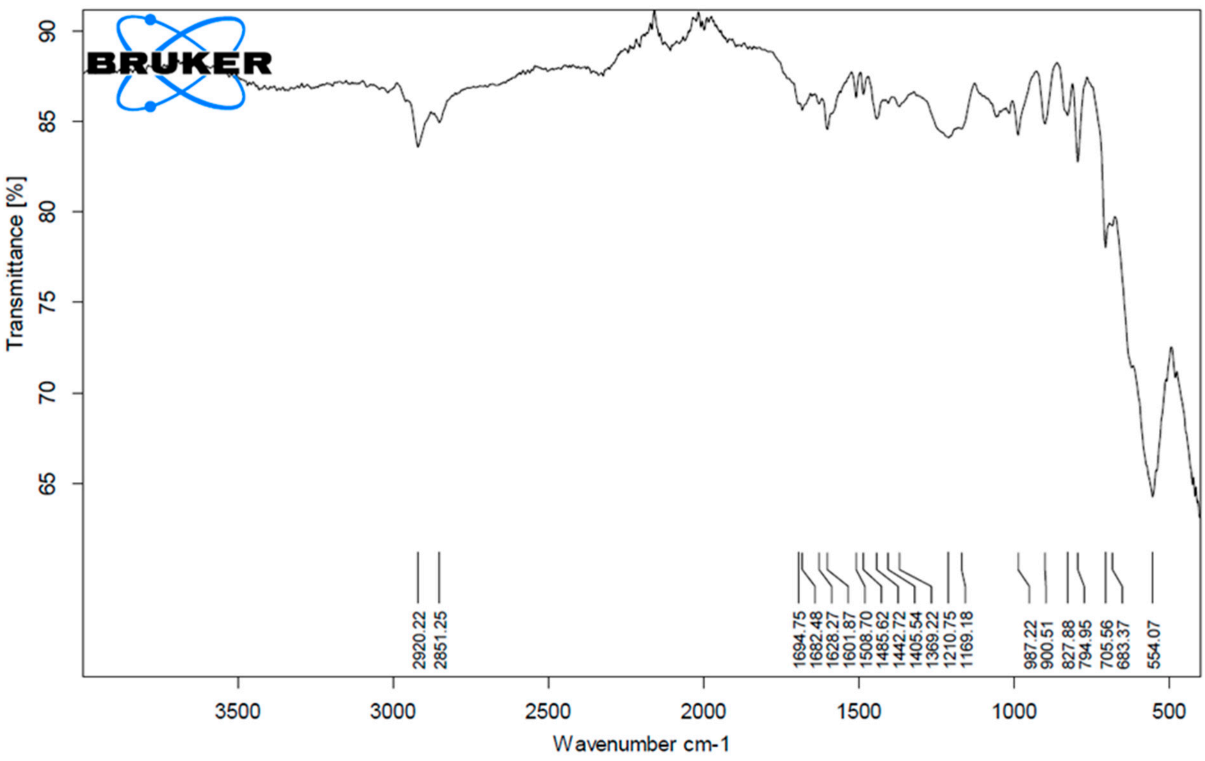

Figure S13. FFT-IR spectrum of MNP-Mal after hydrolysis.

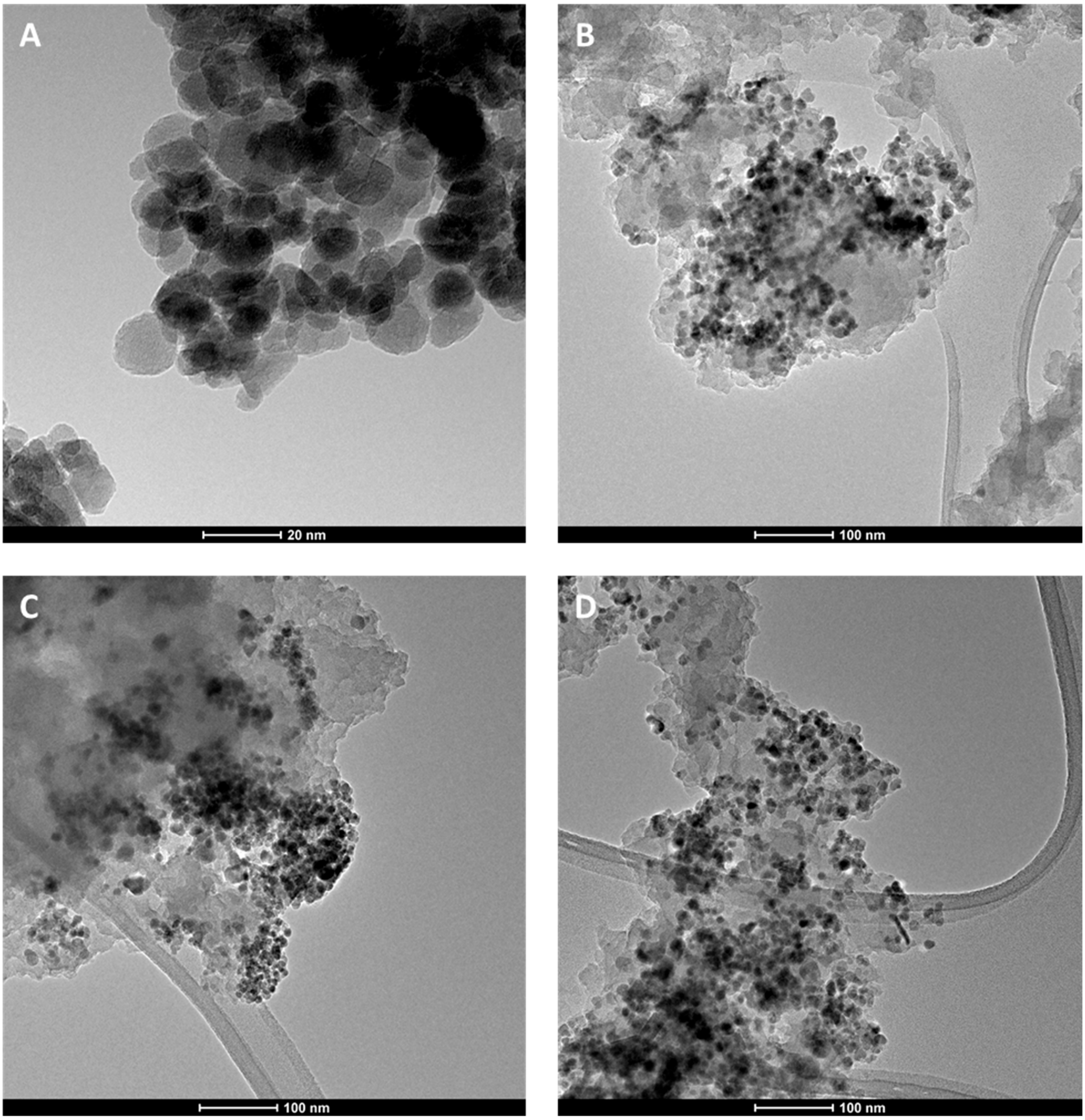

Figure S14. SEM images of (A) Fe_3O_4, (B) MNP-Ac, (C) MNP-Sul, and (D) MNP-Mal.

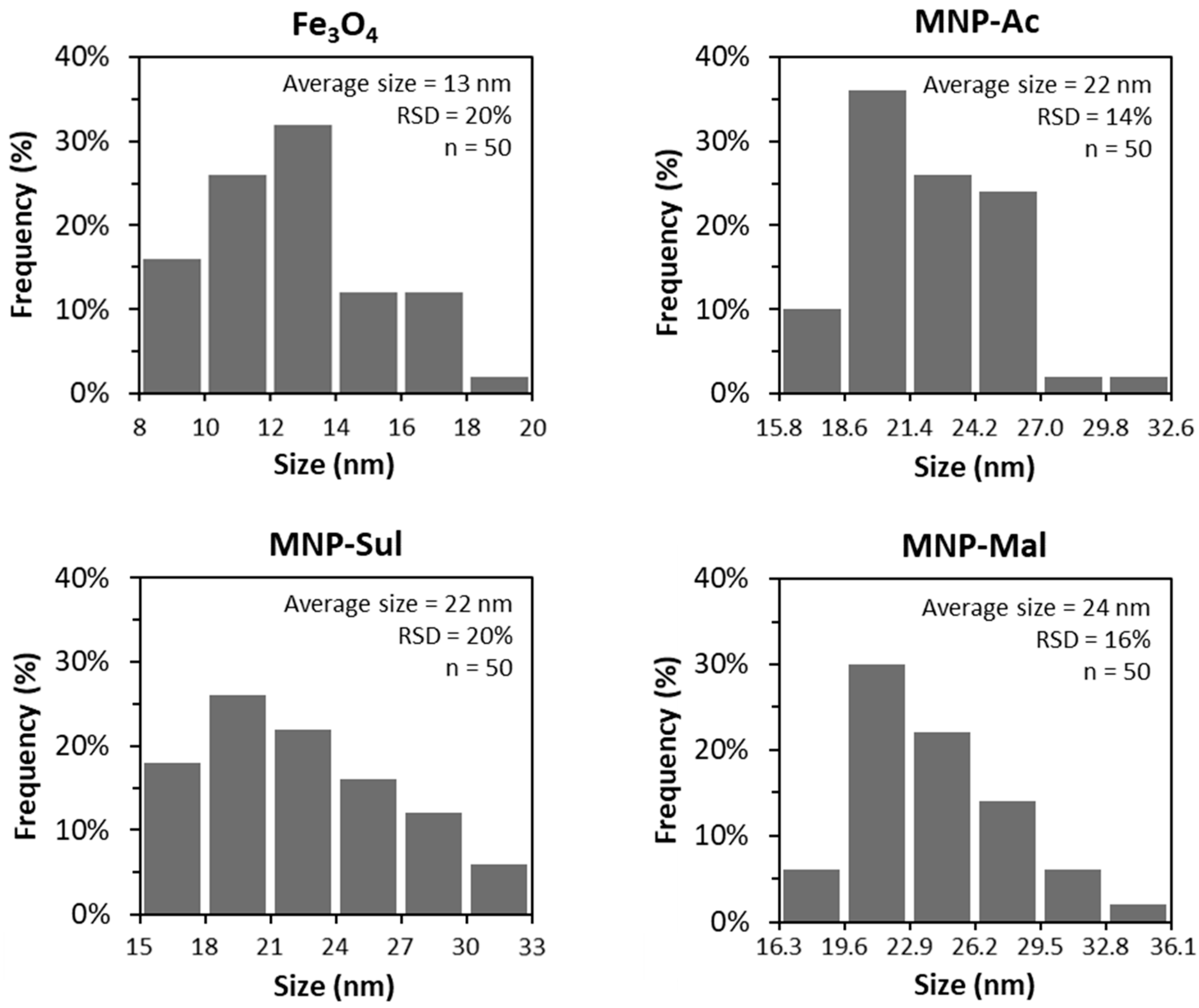

Figure S15. Particle size distribution for obtained MNP. Abbreviations: RSD - relative standard deviation; n - sample size.
